# Advanced control strategy based on hybrid energy storage system for frequency stability of interconnected power system with high renewables penetration

**DOI:** 10.1038/s41598-025-23283-6

**Published:** 2025-11-04

**Authors:** Hossam Hassan Ali, Ahmed Fathy, Mohamed Khamies

**Affiliations:** 1 Electrical Department, Faculty of Technology and Education, Sohag University, Sohag, Egypt; 2 Electrical Engineering Department, College of Engineering, Jouf University, Sakaka, 72388 Saudi Arabia; 3 Department of Electrical Engineering, Faculty of Engineering, Sohag University, Sohag, Egypt

**Keywords:** Frequency control, Renewable energy penetration, Plug-in electric vehicles, Superconducting magnetic energy storage, Cyber security attaks, Electrical and electronic engineering, Energy grids and networks

## Abstract

This paper presents a novel strategy to achieve adjustable frequency stability in hybrid interconnected power systems with high penetration of renewable energy sources (RESs). The considered system incorporates real-world RESs data to emulate practical grid operation, addressing the challenges posed by RESs variability and intermittency more realistically than previous works. The proposed approach integrates a hybrid energy storage systems (HESSs) with load frequency control (LFC) based on a proportional derivative–proportional integral (PD-PI) controller. The HESSs leverage the complementary strengths of plug-in electric vehicles (PEVs) and superconducting magnetic energy storage (SMES) units, with PEVs providing long-term energy balancing and SMES ensuring rapid transient response. The PD-PI controller is applied to simultaneously manage both LFC and HESSs operation, with its parameters optimally tuned using the electric eel foraging optimizer (EEFO) to ensure precise and effective controller. Comparative analyses demonstrate that the PD-PI controller significantly outperforms traditional proportional integral derivative (PID) controllers in maintaining frequency stability under high-RESs penetration and load disturbances. Specifically, the proposed strategy improves system performance by 55% compared to SMES-based PD-PI controllers and by 45% compared to PEVs-based PD-PI controllers. While the approach is most effective in hybrid systems with available PEVs infrastructure and SMES units, its applicability may be limited in power systems lacking such resources or facing large-scale, long-duration disturbances. Furthermore, the effectiveness of the proposed strategy has been validated under cyber attack conditions ,and system parameters variations. Overall, the findings confirm the critical role of the proposed strategy in mitigating frequency fluctuations during periods of high renewable energy penetration, thereby offering a robust solution for the challenges faced by modern power systems.

**Supplementary Information:**

The online version contains supplementary material available at 10.1038/s41598-025-23283-6.

## Introduction

Renewable energy sources (RESs) are being incorporated into power systems to meet the growing energy demand. Additionally, the RESs are installed to minimize the gas emissions resulting from conventional power plants. So, the RES are becoming essential components in the transition towards a sustainable energy future. On the other hand, installing RESs in recent grids via power electronics devices causes harmful effects to these grids. The harmful effects resulted from these electronic devices, which cause high losses of system inertia, especially with the high installation of RESs in grids^1–4^. As a result, the losses of system inertia reduce the system stability and increase the frequency fluctuations. So, the integration of RESs requires advanced technologies and approaches to manage adaptability and ensure consistent energy supply for customers^5–7^. In this regard, huge efforts have been established to reduce the frequency fluctuations and keep the stability of the system.

### Literature review

The load frequency control (LFC) is considered the most common solution to maintain the frequency at nominal standards. In this regard, different control methods have been performed in LFC to maintain a frequency stable at nominal standards^8^. These methods involve optimal control techniques such as sliding mode control^9^, artificial neural network^10^, model predictive control (MPC)^11,12^, robust control techniques^13^, fuzzy control techniques^14^, hybrid controllers that consist of more than one controller as fuzzy based fractional order control^15^, and freedom degree fractional order control with MPC^16^. These strategies effectively addressed LFC problems, however relied on the developer’s expertise and experimentation methods. Additionally, these strategies estimated long time to define their parameters.

Despite significant advances in the development of advanced controllers in the past few decades, PID controllers remain a popular engineering choice due to their easy design and cheap prices. In the presence of system imperfections and nonlinearities (i.e., RESs penetration and load perturbations), the PID controller faces challenges due to the complexity of selecting its parameters, especially with high RESs penetration^17^. In this regard, several researchers applied different structures of improvement to the traditional PID controller such as cascaded controller arrangement (CCA)^18^, a feed forward-feedback arrangement^19^, combination arrangement^20^ and increasing the degree of freedom^21^. Among these methods the CCA is distinguished by simple construction and easy to implement. The reason for applying to the CCA is due to its ability to enhance the outcomes through more tuning parameters. As a result, the CCA design is among the most effective control techniques for boosting system robustness, particularly when disturbances are present in control applications. In this regard several arrangements of the CCA have been applied such as PID-PID^22^, PD-PID^23^, PID-P^24^, fuzzy PI^25^, fuzzy PI-PID^26^, and variable structure MPC based PI^27^. Consequently, this is the authors suggestion for using CCA (i.e. PD-PI) in LFC to improve the traditional PID controller performance.

With the increasing concentration of RESs in the power system, LFCs that rely on diverse control techniques are unable to deal with the nonlinearities caused by high RESs. So, it is essential to install different systems to enhance the system performance and eliminate the RESs bad effects^28–31^. In this regard, energy storage schemes (ESSs) play a crucial role in increasing the efficiency and effectiveness of power systems during high RESs penetrations^32,33^. As a result, different ESSs have been employed to enhance power system execution like fuel cells^34^, super capacitor (SC)^35^, plug electric vehicles (PEVs)^36^, super magnetic energy storage system (SMES)^29^, capacitive energy systems^37^, redox flow batteries (RFBs)^38^. As the RESs penetration increases, significant efforts have been made to utilize the different advantages of these ESSs. In this regard, different studies considered hybrid ESSs (HESSs) to enhance the performance of the power grids during high RESs penetration^39^. The HESSs which applied to enhance the performance of the power grids are EVs and SC^40^, Vanadium- RFBs and SMES^41^, PEVs and FCs^42^. However, these ESSs improved the power grid during high RESs penetration, they have significant limitations. As, it requires a controller to control the power injected or absorbed by the ESSs. Building on the benefits of the HESSs, the authors aim to apply HESSs as RESs penetration increased.

Too far, several optimization techniques have been put forward for identifying the limit values of various control schemes to address system stability problems. Different optimization algorithms have been used to select the optimal parameters of the considered controller in LFC or linked with ESSs such as a novel sooty terns algorithm^12^, multi-verse optimizer^43^, marine predator optimizer algorithm^44^, cheetah optimizer^45^, and skill optimization technique^46^. In this regard, the authors applied electric eel foraging optimizer (EEFO) to select the optimal parameters of the proposed controller in this study^47^.

### Research gap and motivation

In addressing frequency stability challenges, conventional controllers like the I, PI, and PID controllers encounter difficulties in parameter tuning and lack deliberation for doubt, averting system stability. Additionally, other non-cascaded controllers have enabled LFC of power systems; nevertheless, high performance with heavy penetration of RESs was not permitted by these controllers. On the other hand, few studies applied CCA to maintain frequency stability, but their arrangement uses more parameters, and the used optimization techniques cannot select the parameters with precision. Also, the developed algorithms have drawbacks like sluggish convergence during the iterative process, trapping in local optima, and adjustment of governing parameters. In addition, only an insufficient study has observed the influence of high RESs diffusion. Consistently, limited reviews consider the use of the EESs without their hold controllers in power grids with excessive RESs penetration. Nevertheless, these ESSs enhance the power grid performance, but they do not proposal superior solutions for improving the frequency fluctuations. Also, several previously used approaches use traditional ESSs without any control to enhance the performance of their considered systems. This administration highlights the need for a more inclusive analysis of the integration of advanced ESSs alongside robust control strategies to mitigate frequency variations through high RESs penetrations.

This work’s motivation is to avoid the flaws in the previously published methodologies by using an effective strategy to enhance frequency fluctuations during high RESs penetration. The effective strategy depended on applying HESSs (i.e., PEVs with SMES). Furthermore, applying a PD-PI controller to control the supplied or absorbed HESSs. Additionally, utilizing the benefits of the EEFO in selecting the parameters of the proposed controller. The authors considered the output of RESs sources to confirm the superiority of the proposed strategy in enhancing the frequency and mitigating any fluctuations. Additionally, Table 1 explains the distinctions between the present research and the previously stated findings as well as the limitations of the previously mentioned findings.

**Table 1:** The distinctions between the present research and the previously stated findings.

Refs.	Secondary Controller	Optimization method	Considering ESSs	Considering controller for ESSs	RESs penetration
^7^	PID	Enhanced Whale Optimization Algorithm	SMES	×	Low
^18^	TD-TI	quantum chaos game optimizer	EVs	×	Low
^19^	ITDF	imperialist competitive algorithm	×	×	Low
^34^	MDOF-TI ^λ^ D ^µ^ N	Capuchin search algorithm	FC	MDOF-TI ^λ^ D ^µ^ N	Low
^38^	fuzzy PID- TI λ D µ N	crayfish optimization algorithm	RFB	PID	High
^40^	3DOF-TID&FOPID	Transit Search optimization	EVs & SC	FO-PI	Low
^41^	PID	Golden Eagle Optimization	(VRFB & SMES)	×	Low
^44^	2DOF TIDN-TDN	marine predator optimizer algorithm	EVs	TID	High
^48^	FOPIDN+(1 + TD)	Quasi Opposition Arithmetic Optimization Algorithm	RFB& SC	×	Low
This study	PD-PI	Electric eel foraging optimizer	PEVs & SMES	PD-PI	High

### The study contribution

This study proposes the use of HESSs (i.e., PEVs and SMES) to mitigate the frequency instabilities through high RESs penetration. The HESSs depend on the PD-PI controller during their operation in the considered power grid. The HESSs operates with the LFC (i.e., PD-PI controller) to enhance the system performance. Additionally, the EEFO algorithm was used to choose the optimal parameters of the proposed HESSs strategy. Furthermore, the following points summarize this work’s significant contributions:

- A novel hybrid energy storage system (HESSs) integrating PEVs for long-term balancing and SMES for rapid transient support, providing enhanced frequency stability and operational efficiency.
- A realistic high-renewable framework that incorporates actual wind and solar data from Egypt, ensuring practical evaluation of system dynamics under high-RES penetration.
- A cascaded PD-PI controller applied simultaneously to both LFC and HESSs operation—representing the first dual application of this controller—which effectively mitigates frequency deviations in RES-dominated grids.
- Optimal tuning of the PD-PI controller using the Enhanced Exploration and Exploitation Framework Optimization (EEFO), ensuring precise parameter selection and superior control performance.
- Comprehensive robustness validation of the proposed controller against diverse operating challenges, including system parameter variations, different load patterns, and cyber-security attack conditions rarely addressed in previous studies.

### The study organization

The remainder of the paper is structured as follows: Sect. 2 provides the dynamic system description. Section 3 describes the problem and the PD-PI control technique. Then, the EEFO algorithm is explained in Sect. 4, while the proposed strategy based on EEFO methodology is presented in Sect. 5. The simulation outcomes and disputation of different scenarios are presented in Sect. 6. Finally, Sect. 7 introduces the study’s conclusions and future recommendations.

## Description of dynamic system

**Fig. 1:**
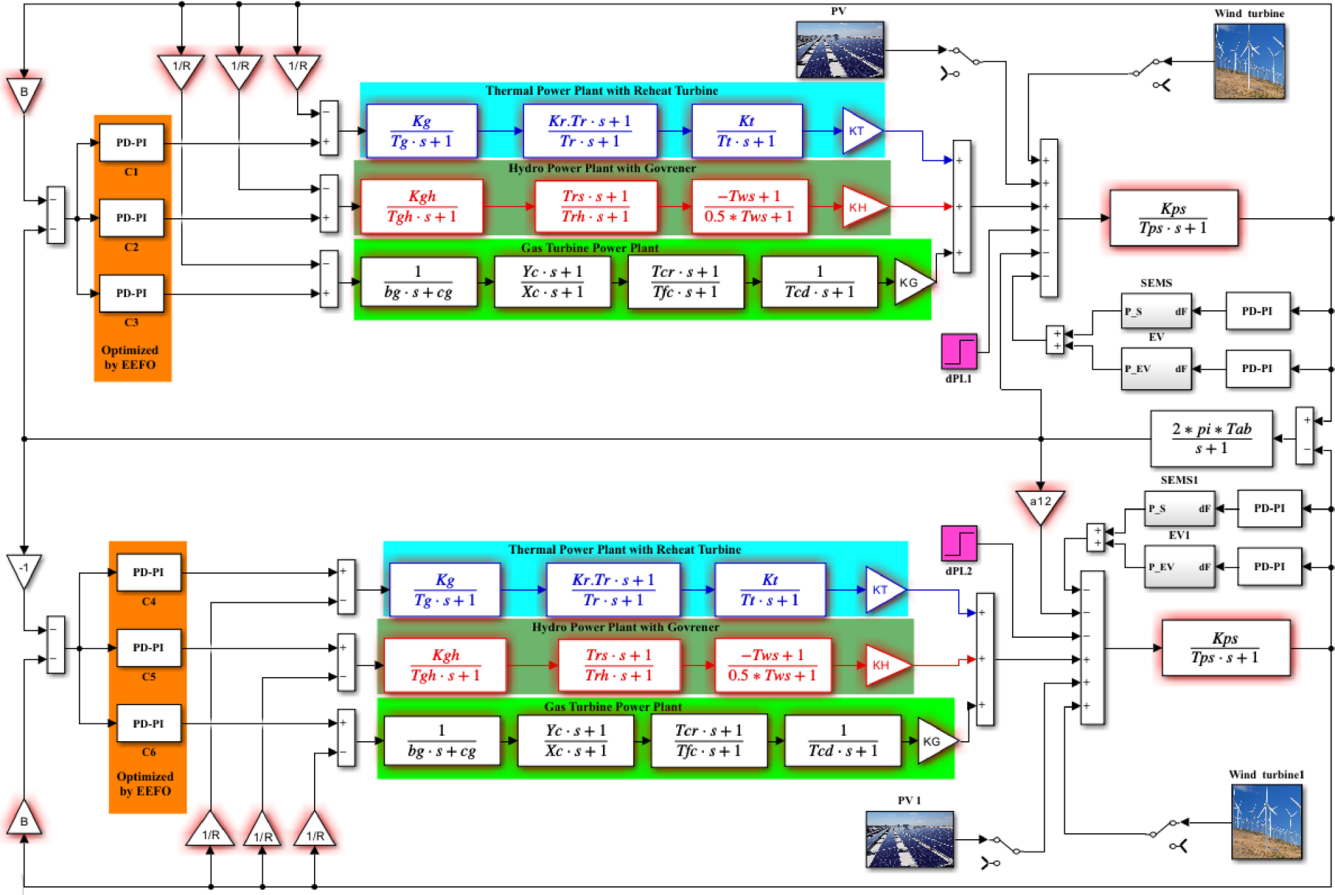
Simulink model of two interconnected system.

This study discusses hybrid power grid (HPG) that incorporates RESs and ESSs technology. The PD-PI is employed in LFC to achieve power grid stability. Each region of the HPG includes three traditional generation plants: a gas plant, a hydroelectric plant, and a reheat thermal plant. Each region of the HPG has an overall rated capacity of 2000 MW. The thermal power plant contributes 1087 MW, obtaining the biggest amount of electrical power; the gas turbine of 262 MW and hydropower capacity of 653 MW come in second and third, respectively. Figure 1 displays the description of the power grid in the Simulink model. The input signal for the proposed controller is the area control error (ACE), while each generation unit’s output signal serves as a secondary control action to obtain more active power for better performance. Table S1 in Supplementary clears the parameters of the two identical regions of the electrical grid under study: Eqs. (1) and (2) offer formulas for computing the ACEs in each area^10^.

1 $$\:{ACE}_{1}={\varDelta\:P}_{tie12}+B{\varDelta\:f}_{1}$$

2 $$\:{ACE}_{2}={\varDelta\:P}_{tie21}+B{\varDelta\:f}_{2}$$

where $\:{\varDelta\:P}_{tie12}$ symbolizes the power flow that flows from region-1 to region-2, $\:{\varDelta\:P}_{tie21}$indicates the power flow that flows from region-2 to region-1, $\:B$ represents the bias frequency factor, $\:{\varDelta\:f}_{1}$ and $\:{\varDelta\:f}_{2}$indicate the frequency deviations in region-1 and region-2, respectively.

### Wind power plant model

The following is a description of the output power obtained by real wind turbine^49^:

3 $$\:{P}_{wt}=\:\frac{1}{2}\rho\:{A}_{T}{v}_{w}^{3}{C}_{p}\left(\lambda\:,\beta\:\right)$$

where $\:{v}_{w}$, $\:{A}_{T}$ and $\:\rho\:$ indicate the velocity of wind, the swept area, and the air density, respectively, $\:\beta\:$ and $\:\lambda\:$ represent pitch angle of the blade and ratio of tip-speed, respectively. The following formula expresses the power coefficient ($\:{C}_{p}$), also known as the rotor blades coefficient^50,51^:

4 $$\:{C}_{p}\left({\lambda\:}_{i},\beta\:\right)=0.5\left({\lambda\:}_{i}-0.022{\beta\:}^{2}-5.6\right){\times\:e}^{-0.17{\lambda\:}_{i}}$$

5 $$\:{\lambda\:}_{i}=\frac{3600\times\:R\:}{1609\times\:\lambda\:}\:,\:\:\lambda\:=\frac{\omega\:\times\:R}{{v}_{w}}\:$$

where $\:R$ and $\:\omega\:$ denote the blade length and angular velocity.

**Fig. 2:**
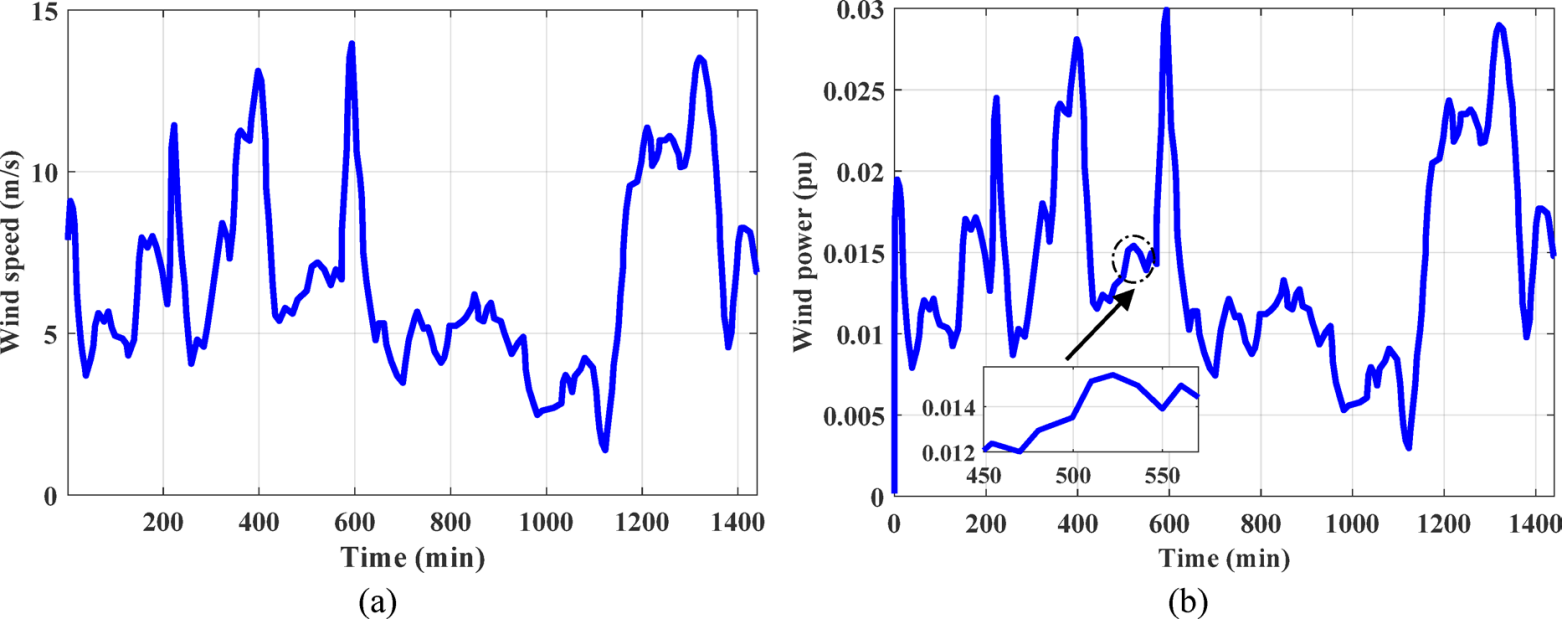
The electrical power generated by real wind farm^17^.

In both regions under examination, wind power plants have a high penetration rate as one of the main RESs. wind farm with rated capacity of 85 MW has been used and linked into the power grid under investigation^17^. Furthermore, the whole bulk employed in this study is ten Zafarana wind farms, with a total capability of 850 MW. The transfer function used in arithmetic model of the wind induction generator has a gain of unity and time constant of 0.3 s. The actual wind speed recorded in the Jabal al-Zeit area in the Red Sea Governorate in Egypt and the actual random output power from the wind power station in a real platform are shown in Fig. 2.

### The model of solar power plant

**Fig. 3:**
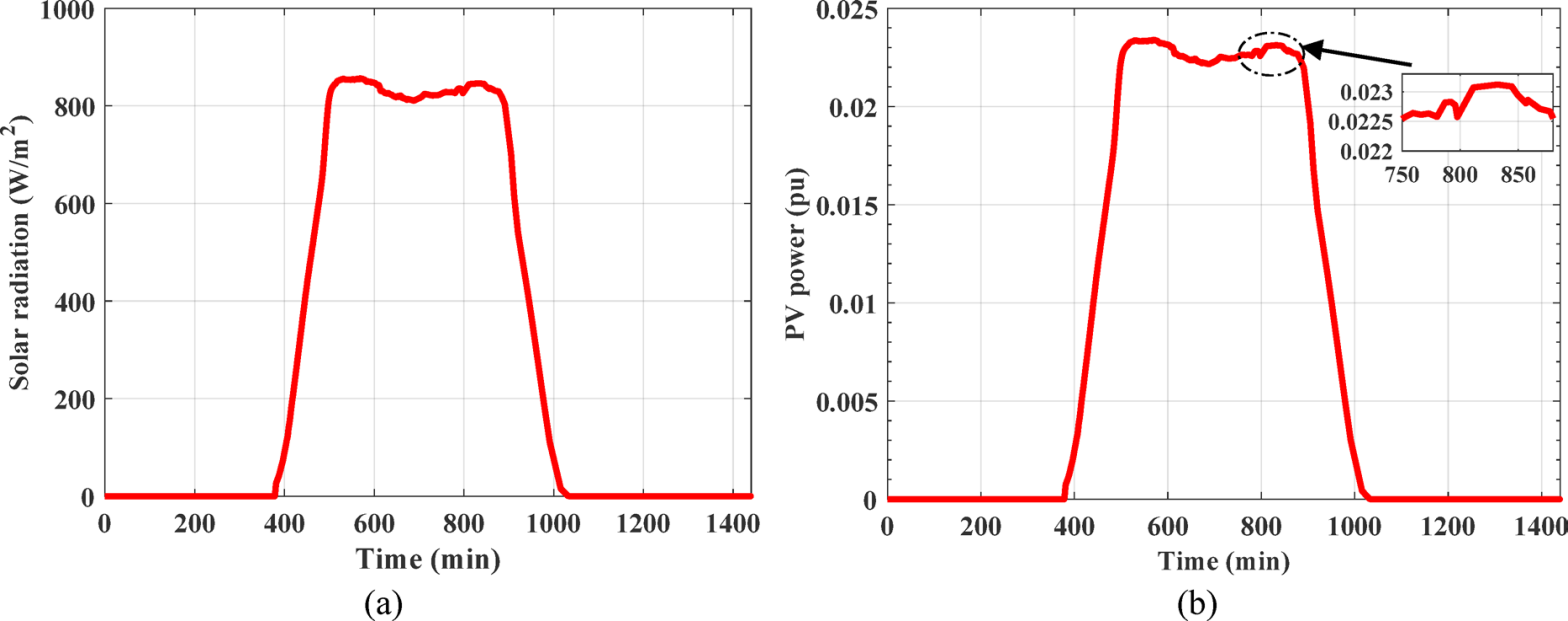
The produced power from real PV plant^17^.

The irradiation intensity, surface area, and temperature of the surrounding environment influence the power output of PV generating unit. To determine the electrical power extracted from the PV module, the following Eq. is used^52^:

6 $$\:{P}_{PV}={\eta\:}_{sc}{\tau\:}_{g}{\alpha\:}_{sc}RA\:[1-{{\upmu\:}}_{sc}({T}_{sc}-{T}_{r}\left)\right]$$

where $\:A$, $\:{\eta\:}_{sc}$, $\:{\alpha\:}_{sc}$, and $\:{\mu\:}_{sc}$ denote the overall area of solar cell, efficiency of solar cell, absorptivity of solar cell, and thermal factor of PV cell efficiency, respectively. The terms $\:R$ and $\:{\tau\:}_{g}$ symbolize solar radiation and glass permeability, respectively, $\:{T}_{r}$ and $\:{T}_{sc}$ indicate reference temperature and cell temperature, respectively. PV plant with 50 MW capacity is considered and linked to the investigated power grid^17^. Solar radiation recorded in the Benban area in Aswan Governorate in Egypt and the power produced by real PV plant is illustrated in Fig. 3.

### Plug in electric vehicles model

**Fig. 4:**
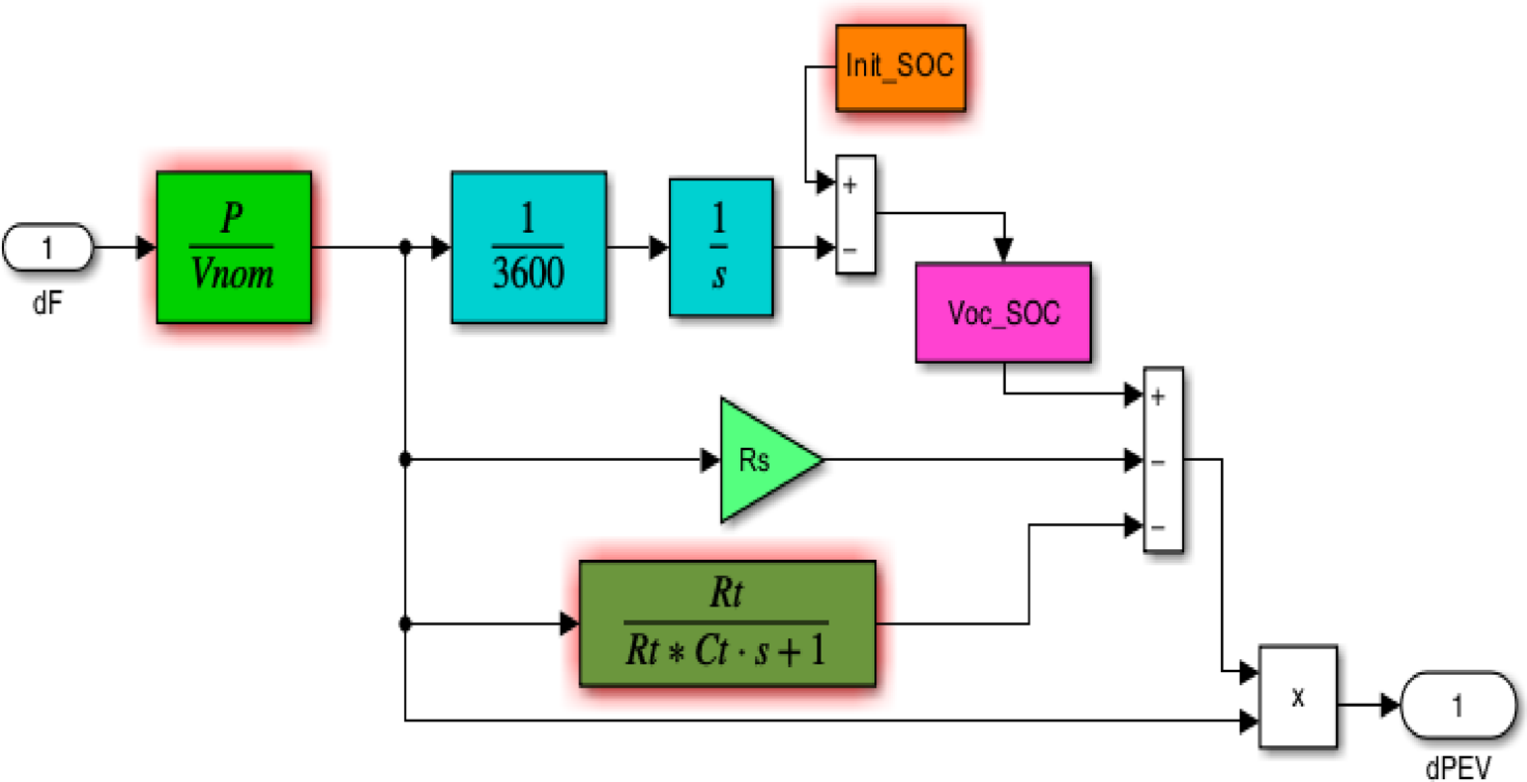
The PEVs model in Simulink^55^.

Numerous works presented storage technology of PEVs to reduce the imbalance between production power and load demand when RESs are considered. By connecting PEVs to the electrical grid, the superior potential utilization of electrical energy between the power system and consumers is achieved. As a result, using comparable PEV with different inverter ability allows control the model behavior of PEV^53,54^. Figure 4 illustrates the appropriate PEV design for the LFC investigation; this model aims to simulate the performance of PEV battery. It can calculate the total power required to charge or discharge battery under control. In this model, the voltage of an open-circuited battery is represented by $\:{V}_{oc}$. The voltage of an EV is contingent upon the state of charge ($\:SOC$) of its batteries. To simulate ohmic losses and battery transients, parallel RC network with series resistance is included in the model^21^. The Nernst voltage can be calculated as follows:

7 $$\:{V}_{oc\_SOC}={V}_{nom}+S\frac{RT}{F}\text{ln}\left(\frac{SOC}{{C}_{nom}-SOC}\right)$$

where $\:{C}_{nom}$ and $\:{V}_{nom}$ represent the nominal values of battery capacity and battery voltage, respectively, $\:S$ and $\:T$ stand for the parameters’ sensitivity of Nernst voltage and temperature, respectively. The terms $\:R$ and $\:F$ symbolize gas and Faraday constants, respectively. The size of PEV is selected based on the battery capacity, the maximum charging and discharging capacity for each vehicle connected to the grid, and the number of potential connected vehicles. Considerable adjustment of frequency and a decrease in total load variations are rendered achievable by PEV penetration^55^.

### Superconducting magnetic energy storage model

**Fig. 5:**
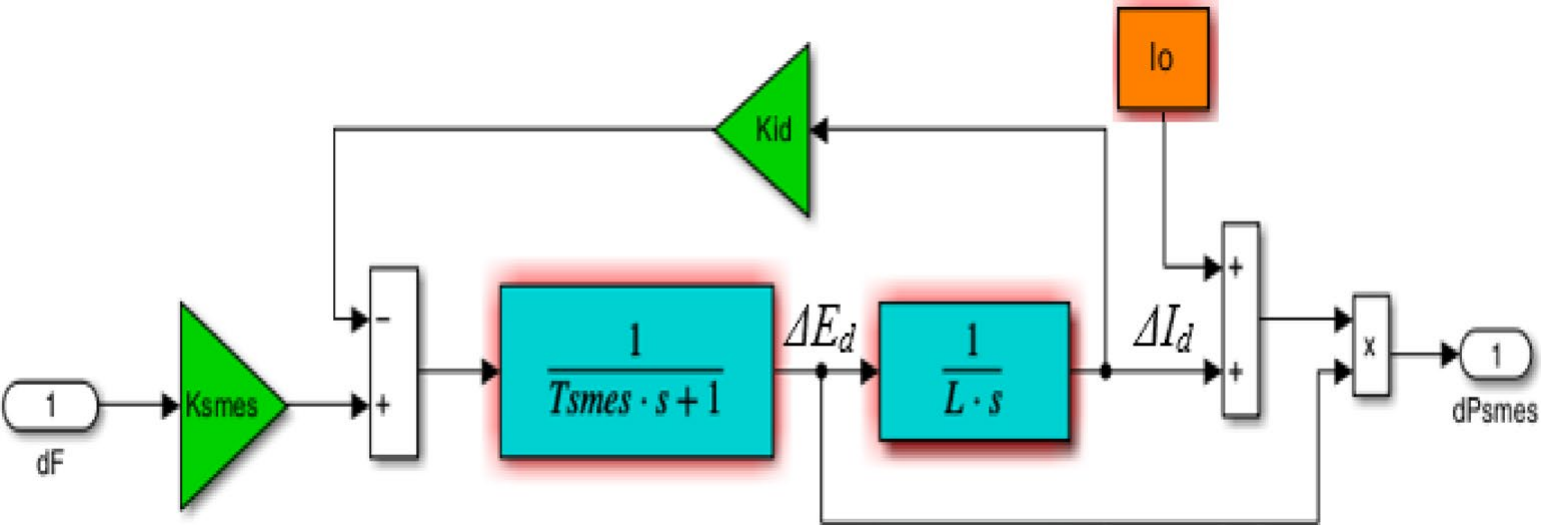
The SMES model in Simulink^57^.

The SMES units have an extreme superconducting inductance that allows them to absorb surplus real power when load decreases and injects real power as a substitute during increasing load. The SMES releases the stored energy into the grid through the power conversion system in response to an abrupt shift in the load demand. It helps in increasing the energy generated so that the system requires less time to be stabilized, the block diagram of SMES is shown in Fig. 5. Compared to other ESSs, SMES devices exhibit higher efficiency, longer working lifetimes, and faster reaction times. Fast power grid disruptions can be handled by the SMES, improving frequency response regulation^56,57^. The power deviation of SMES can be calculated as,

8 $$\:{\varDelta\:P}_{smes}=Io.{\varDelta\:E}_{d}+\varDelta\:{I}_{d}.\varDelta\:{E}_{d}$$

9 $$\:\varDelta\:{I}_{d}=\frac{1}{SL}\varDelta\:{E}_{d}$$

10 $$\:\varDelta\:{E}_{d}=\frac{1}{S{T}_{Smes}+1}\left({K}_{smes}.\varDelta\:F-{K}_{id}.\varDelta\:{I}_{d}\right)$$

where $\:Io$ and $\:{\varDelta\:E}_{d}$denote the current’s nominal value and voltage deviations, respectively, $\:\varDelta\:{I}_{d}$ refers to the current’s deviations, $\:L$ represents the coil inductance, and $\:{K}_{id}$is the gain of the feedback loop for inductor deviation. The terms $\:{T}_{Smes}$, $\:{K}_{smes}$, and $\:\varDelta\:F$ symbolize the time constant, gain of SMES, and frequency deviation, respectively. The SMES size is selected based on its power rating, energy capacity, charging and discharging time, and system requirements. When SMES injects power very instantly, it enhances frequency regulation and dampens fluctuations rapidly.

## The PD-PI control arrangement

**Fig. 6:**
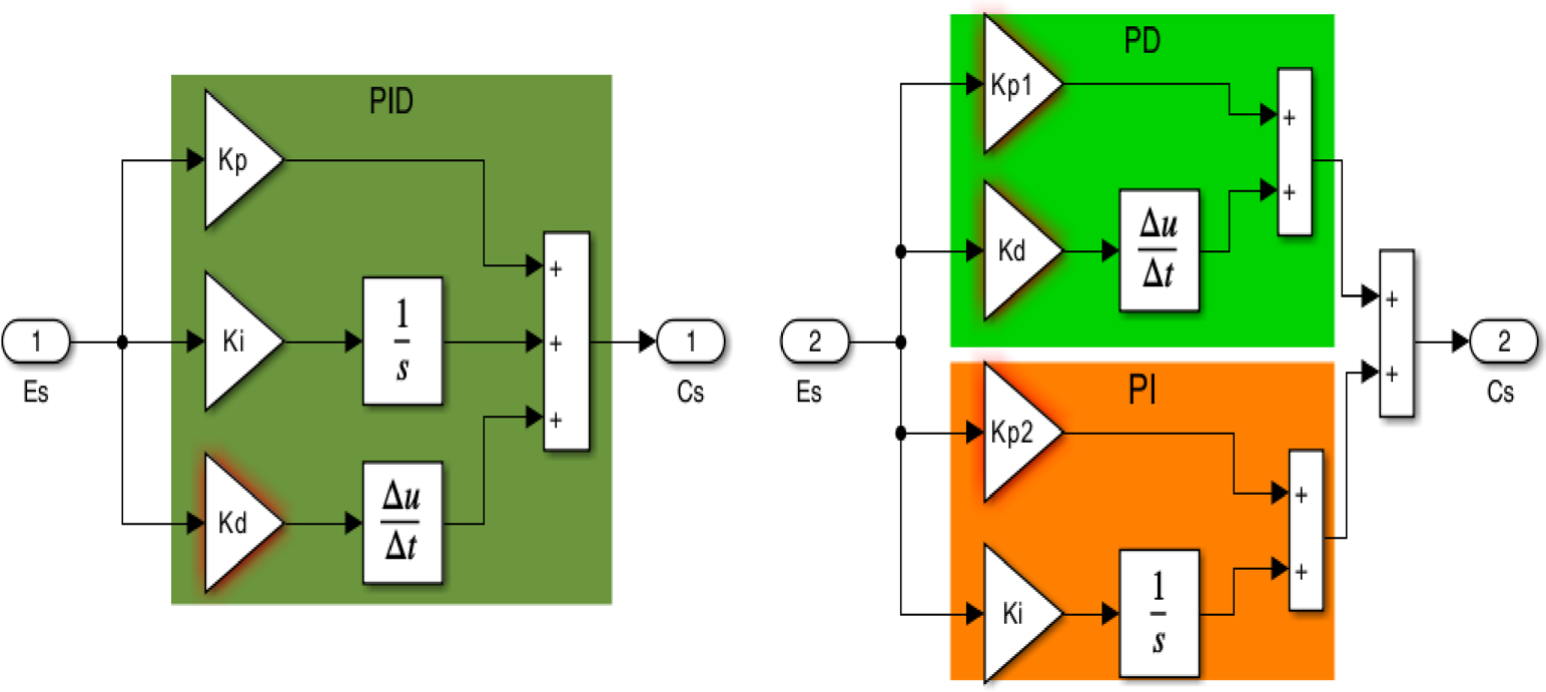
The structure of PID and proposed PD-PI controllers.

Due to the topic issues in the power grid under study which is represented in high penetration rates of RESs in both regions and various relevant fluctuating load patterns, it is imperative to build a dependable controller to reduce oscillations and increase system stability. Thus, to address the oscillations that resulted from the earlier issues, this work considers developing PD-PI controller designed via EEFO algorithm. The controller process’s main goal is reducing the impact of the primary oscillations and improving the quality of the output. The structure of the PID and proposed PD-PI controllers are displayed in Fig. 6, the proposed controller has only four parameters that are calculated by EEFO method. Advantages of PD-PI Over Traditional PID as:

- Improved disturbance rejection & setpoint tracking separation.
- PID controllers combine proportional (P), integral (I), and derivative (D) actions into a single structure, which means the same gains affect both disturbance rejection and setpoint tracking.
- PD-PI controllers decouple these two functions: PD part (Proportional-Derivative) primarily handles setpoint tracking (improve transient response). PI part (Proportional-Integral) primarily handles disturbance rejection (steady-state error elimination).
- Result More flexible tuning for different control objectives.
- Reduced Overshoot in Setpoint Changes.

For more clear around the derivative (D) action in the PD part helps dampen overshoot when tracking reference changes, whereas in a standard PID, the derivative term affects both tracking and regulation, sometimes causing conflicts.

The transfer functions of PID and PD-PI controllers can be expressed as,

11 $$\:{TF}_{PID}=\frac{C\left(s\right)}{E\left(s\right)}={K}_{p}+\frac{{K}_{i}}{S}+{K}_{d}S$$

12 $$\:{TF}_{PD-PI}=\frac{C\left(s\right)}{E\left(s\right)}={K}_{p1}+{K}_{d}S+{K}_{p2}+\frac{{K}_{i}}{S}$$

In Eq. (11) the $\:{K}_{p}$, $\:{K}_{i}$,and $\:{K}_{d}$ are the parameters of PID controller, while the PD-PI parameters are $\:{K}_{p1}$_,_ $\:{K}_{d}$_,_ $\:{K}_{p2}$, and $\:{K}_{i}$ as given in Eq. (12). Where *K*_*min*_ ≤ *K* ≤ *K*_*max*_, *K*_*min*_ represents the minimum bound which is assigned as zero, while *K*_*max*_ indicates the maximum bound and assigned as 10. The integral time absolute error ($\:ITAE$) is used as the fitness function to be mitigated, it can be calculated via multiplying the time term by the integral of the absolute error as follows:

13 $$\:\text{M}\text{i}\text{n}\text{i}\text{m}\text{i}\text{z}\text{e}\:ITAE=\text{m}\text{i}\text{n}\text{i}\text{m}\text{i}\text{z}\text{e}\left(\underset{0}{\overset{{T}_{sim}}{\int\:}}\left(\:\left|{\varDelta\:f}_{1\:}\right|\:+\:\left|{\varDelta\:f}_{2\:}\right|\:+\:\left|{\varDelta\:P}_{tie\:}\right|\right).\text{t}\:\:dt\right)$$

where $\:{T}_{sim}$ indicates the total simulation time and $\:dt$ describes the time of simulation.

## Electric eel foraging optimizer

### Inspiration

The EEFO is presented by *Zhao et al.*^47^, it has considered a predator as it contains thousands of electrical cells that can store electrical energy. Adult eels can generate a voltage of 300 to 800 V to hunt their prey, and it can be powerfully discharged into freshwater fish. With their weak eyesight, eels often create about 10 V to detect and navigate towards prey. Eels efficiently monitor and identify the location of swiftly moving prey by exploiting the feedback given by these electrical impulses. When eels locate their target, they stun them by quickly emitting a stronger electric charge. The highly developed mode of defense and communication that eels master is done by using the electric charge. Furthermore, mild electric discharge is utilized for mutual communication and large electric charges are employed as a weapon of defense against an adversary.

### Interacting

Eels swim and agitate with each other when they come across a group of fish. After that, the eels commence to swim in a massive, electrified circle to catch many small fish within it. Within EEFO, every electric eel represents a potential solution, with the targeted prey being the best nominee solution attained thus far in every phase. Based on the intelligence of each other’s locations, the relationship indicates that each eel collaborates with others. This conduct may be thought of as the global exploration stage. An electric eel may communicate with any other eel selected at random from the population by employing the position data of every member in the community. Comparing the distinction between a randomly selected eel and the population center is necessary to update an eel’s location. Eels churn, or move randomly in various directions, as a kind of social interaction. This churn is illustrated by the following Eqs.:

14 $$\:\left\{\begin{array}{c}\left\{\begin{array}{c}{v}_{i}\left(t+1\right)={x}_{j}+C\times\:\left(\stackrel{-}{x}\left(t\right)-{x}_{i}\left(t\right)\right)\:\:{p}_{1}>0.5\\\:{v}_{i}\left(t+1\right)={x}_{j}+C\times\:\left({x}_{r}\left(t\right)-{x}_{i}\left(t\right)\right)\:\:{p}_{1}\le\:0.5\end{array}\right.\:fit\left({x}_{j}\right(t\left)\right)<fit\left({x}_{i}\right(t\left)\right)\\\:\left\{\begin{array}{c}{v}_{i}\left(t+1\right)={x}_{j}+C\times\:\left(\stackrel{-}{x}\left(t\right)-{x}_{i}\left(t\right)\right)\:\:{p}_{\begin{array}{c}\:\\\:2\end{array}}>0.5\\\:{v}_{i}\left(t+1\right)={x}_{j}+C\times\:\left({x}_{r}\left(t\right)-{x}_{i}\left(t\right)\right)\:\:{p}_{2}\le\:0.5\end{array}\right.\:fit\left({x}_{j}\right(t\left)\right)\ge\:fit\left({x}_{i}\right(t\left)\right)\:\end{array}\right.$$

15 $$\:C=n\times\:d$$

16 $$\:\stackrel{-}{x}\left(t\right)=\frac{1}{2}\sum\:_{i=1}^{n}{x}_{i}\left(t\right)$$

17 $$\:{x}_{r}=low+r\times\:\left(up-low\right)$$

where $\:t$ refers to the current iteration, $\:{x}_{j}$, $\:n$ and $\:d$ denote randomly selected eel’s location within the existing population, population size, and dimensions problem, respectively. The terms $\:r$, $\:{p}_{1}$ and $p_2$ represent the random numbers within (0,1), $\:fit\left({x}_{i}\right(t\left)\right)$ denotes the *i*^th^ electric eel’s applicant position’s fitness, $\:low$ and $\:up$ indicate the lower and upper limits, respectively.

### Resting

A resting zone is established in the area where any one dimension of an eel’s location vector is projected onto the principal diagonal in the search space to improve the search efficiency. Consequently, before appealing in resting behavior, an eel locates its resting posture inside its resting region as,

18 $$\:Z\left(t\right)=Low+\left(\frac{{x}_{rand\{n}^{rand\{d}\:\{t-{Low}^{rand\{d}}{{{Up}^{rand\{d}-Low}^{rand\{d}}\right)\times\:\left(Up-Low\right)$$

19 $$\:{R}_{i}\left(t+1\right)=Z\left(t\right)+\left(2\times\:\left(e-{e}^{\frac{t}{{T}_{max}}}\right)\times\:\text{sin}\left(2\pi\:{r}_{1}\right)\right)\times\:\left|Z\left(t\right)-{x}_{prey}\left(t\right)\right|$$

where $\:{T}_{max}$, $\:{r}_{1}$, and $\:{x}_{prey}$ represent the maximum iteration, random numerals within rang (0,1) and the location vector of the most optimal solution found thus far. While the term $\:\left|Z\left(t\right)-{x}_{prey}\left(t\right)\right|$ indicate the resting area rang. Eels will transfer to the specified resting area after it has been identified. In this case, an eel optimizes its location towards the resting area based on its current resting location there. This behavior can be represented as follows:

20 $$\:{v}_{i}\left(t+1\right)={R}_{i}\left(t+1\right)+n\times\:{(R}_{i}\left(t+1\right)round\left(rand\right)\times\:{x}_{i}\left(t\right))$$

### Hunting

Typically, eels swim in synchrony to form a wide circle and encircle their prey when they come to find it. By using low electric discharges for members, they cooperate and contact with their others regularly in the meantime and the electrified area is designated as hunting area. Because of this conduct, the prey starts to scamper around the hunting region, it becomes terrified and abruptly moves from its present location to various regions of the hunting region. Following the identification of the hunting region, an eel starts to hunt in the region, updating its location in response to the prey’s changed location. The hunting behavior can be expression as follows:

21 $$\:{H}_{prey}\left(t+1\right)={x}_{prey}\left(t\right)+\left(2\times\:\left(e-{e}^{\frac{t}{{T}_{max}}}\right)\times\:\text{s}\text{i}\text{n}\left(2\pi\:{r}_{2}\right)\right)\times\:\left|\stackrel{-}{x}\left(t\right)-{x}_{prey}\left(t\right)\right|$$

22 $$\:\eta\:={e}^{\frac{{r}_{3}(1-t)}{{T}_{max}}}\times\:\text{c}\text{o}\text{s}\left(2\pi\:{r}_{3}\right)$$

23 $$\:{v}_{i}\left(t+1\right)={H}_{prey}\left(t+1\right)+\eta\:\times\:{(H}_{prey}\left(t+1\right)-round\left(rand\right)\times\:{x}_{i}\left(t\right))$$

where $\:{r}_{2}$ and $\:{r}_{3}$ refer to random numerals within (0,1), $\:{H}_{prey}$ and $\:{x}_{prey}$ denote the prey location and the prey selected for hunting, respectively, and $\:\eta\:$ refers to the curling parameter.

### Migrating

When eels see prey, they usually transfer from their resting area to their hunting area. The eels’ migration behavior can be expressed as follows:

24 $$\:{v}_{i}\left(t+1\right)={-r}_{4}\times\:{R}_{i}\left(t+1\right)+{r}_{5}\times\:{{H}_{r}\left(t+1\right)-L\times\:(H}_{r}\left(t+1\right)-{x}_{i}\left(t\right))$$

25 $$\:{H}_{r}\left(t+1\right)={x}_{prey}\left(t\right)+\left(2\times\:\left(e-{e}^{\frac{t}{{T}_{max}}}\right)\times\:\text{s}\text{i}\text{n}\left(2\pi\:{r}_{2}\right)\right)\times\:\left|\stackrel{-}{x}\left(t\right)-{x}_{prey}\left(t\right)\right|$$

where $\:{r}_{4}$ and $\:{r}_{5}$indicate random numerals within (0,1), $\:{H}_{r}$and $\:L$ represent the regarded as any location inside the hunting region and levy flight function, respectively.

### Moving from exploration to exploitation stage

**Fig. 7:**
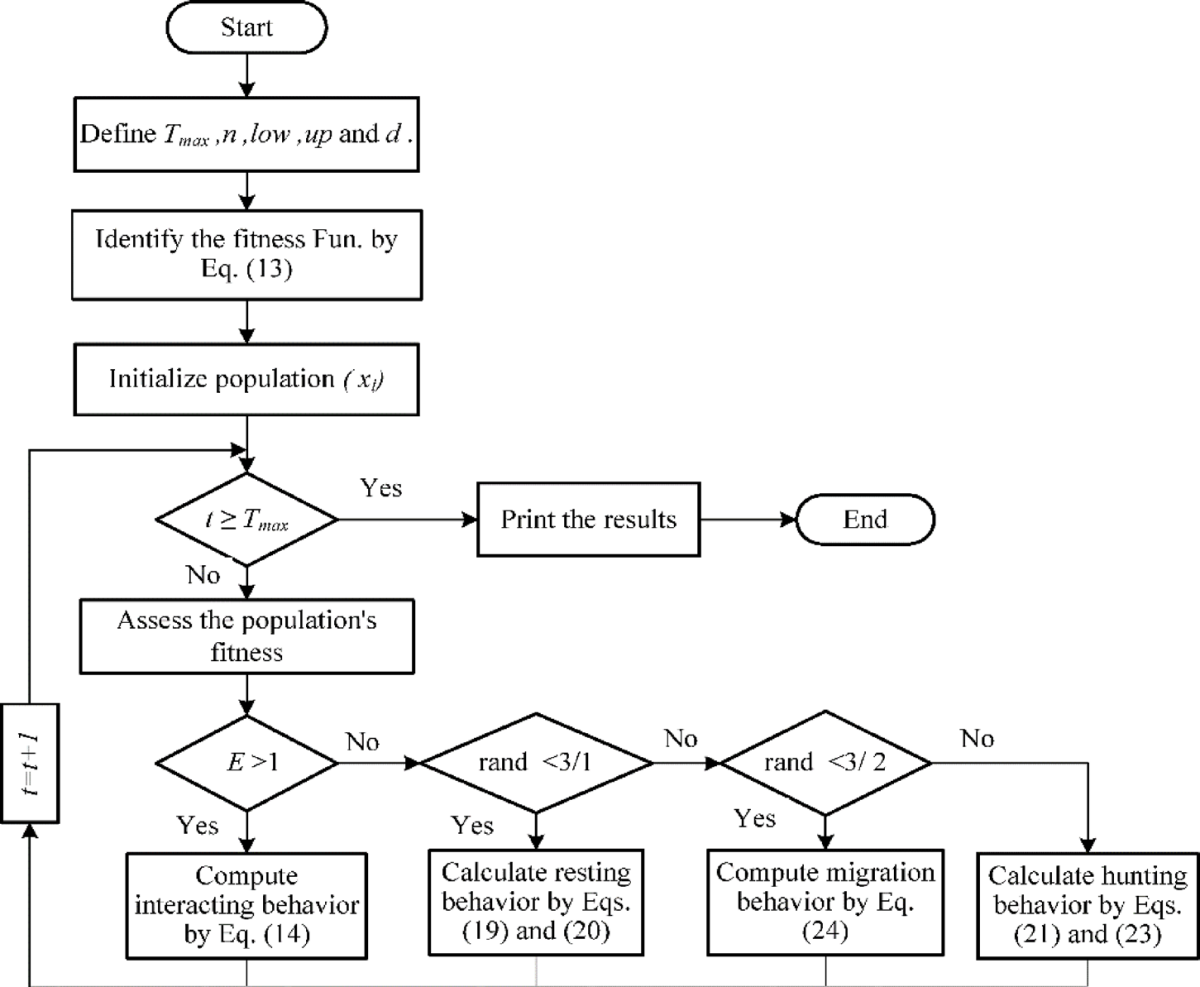
The EEFO flow chart.

To enhance the optimization efficiency of the algorithm, EEFO bases its search behaviors on an energy coefficient that can control the shift from exploration and exploitation. The energy coefficient can be described as,

26 $$\:E\left(t\right)=4\times\:sin\left(1-\frac{t}{{T}_{max}}\right)\times\:\text{ln}\frac{1}{{r}_{6}}$$

where $\:{r}_{6}$ refers to random numeral within (0,1). As the number of iterations increases, the energy coefficient value decreases. The exploration phase is performed when *E(t)* > 1, the eels conduct universal search throughout the variable area by interactive activity. While the exploitation phase is performed when *E(t)* < 1, in this stage the eels typically engage in local search in sub-areas through resting, hunting activities or migration. The flow chart of EEFO is displayed in Fig. 7.

## The proposed strategy based on EEFO methodology

**Fig. 8:**
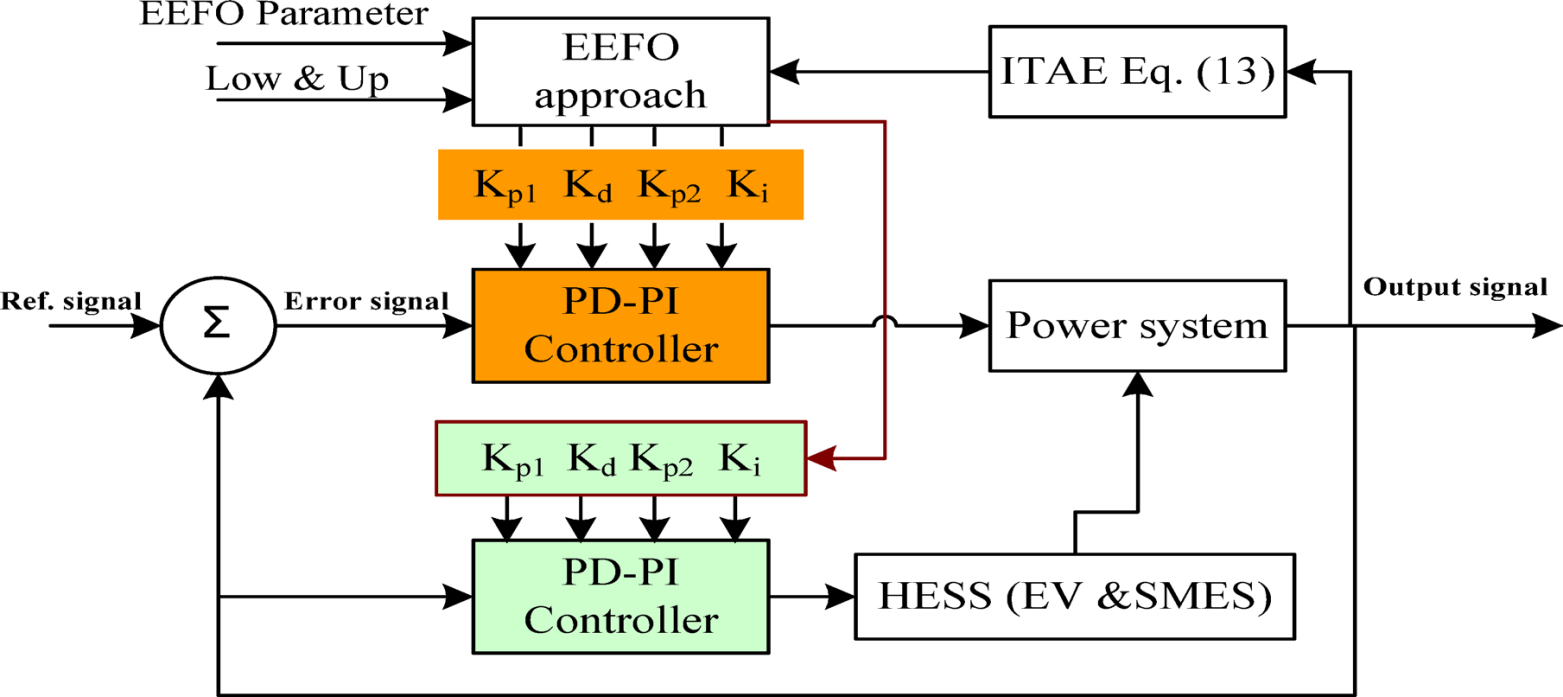
The process of selecting the optimal parameters of the PD-PI controller.

The EEFO is responsible for determining the optimum PD-PI controller parameters, which lead to a minimal ITAE function. The controller relies on the error signal, which represents the difference between the reference signal and the system output signal. The controller supplies the power system with the required control signal to reduce the frequency deviations and power flow in the interconnection line due to load fluctuations. The process of selecting the optimal parameters of PD-PI controller based on EEFO algorithm is presented in Fig. 8, and the steps of EEFO-based strategy provided in algorithm 1.

**Algorithm 1:**
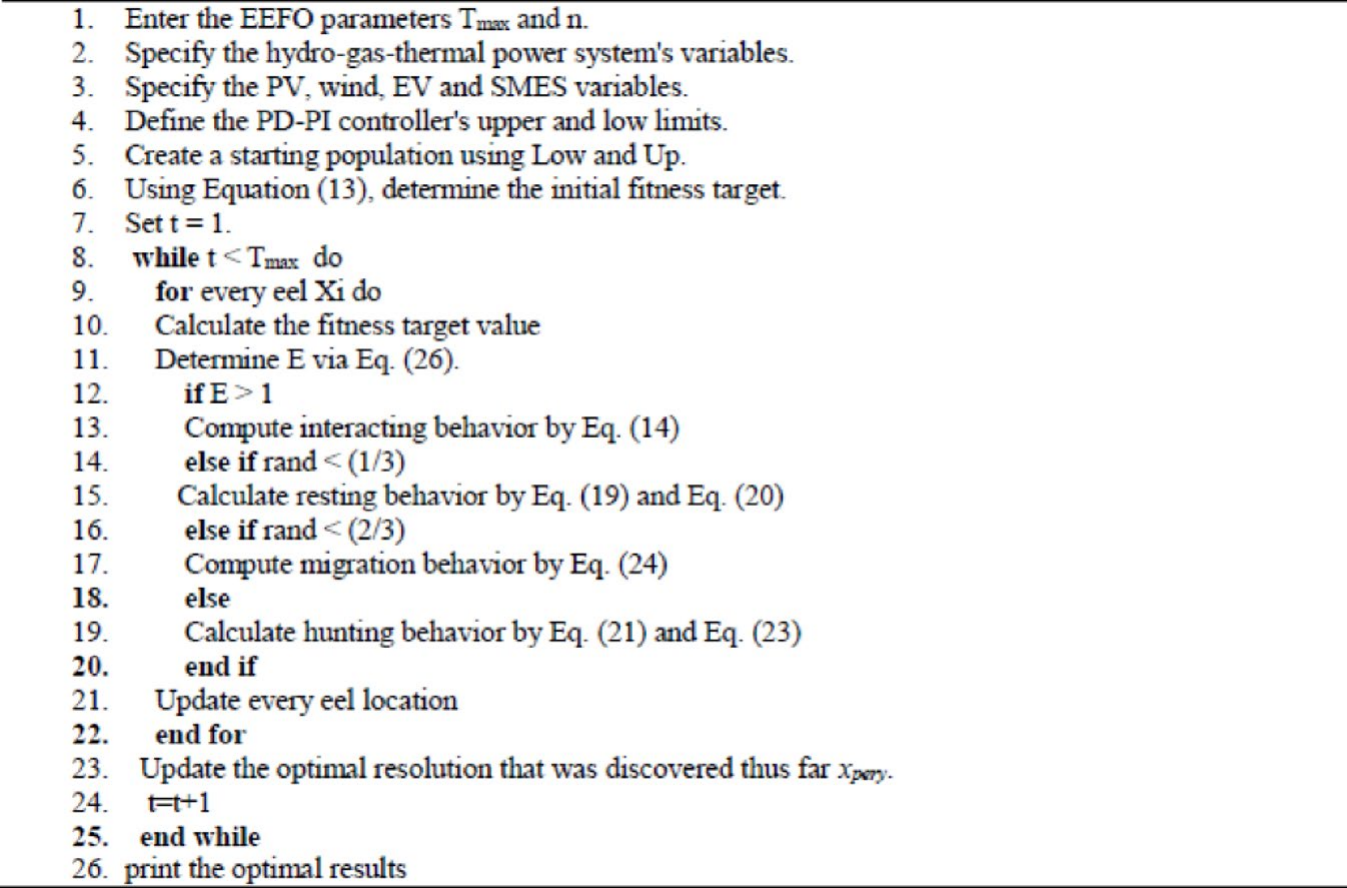
Steps of EEFO-based proposed strategy.

## Results and discussions

**Fig. 9:**
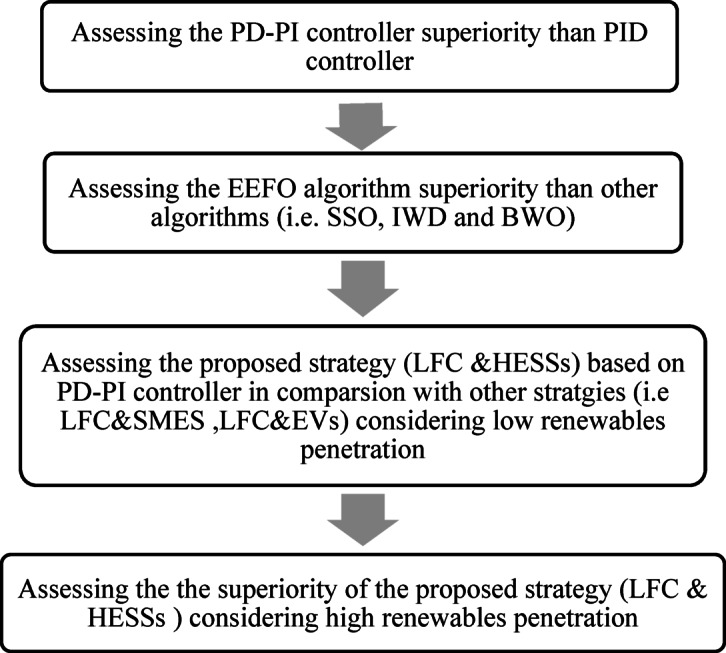
The considered cases according to scenarios for this study.

In this study, the effectiveness of the proposed strategy (HESSs &LFC) based on the PD-PI controller is examined to vanish the frequency violation as well as the exchanged power deviation of a HPG during high renewable power penetration and various demand variation. The proposed strategy controller’s parameters are tunned via EEFO algorithm. Many operating conditions have been considered to validate the superiority of the proposed PD-PI controller in LFC and the proposed strategy. Figure 9 shows the considering cases of validating the proposed PD-PI controller in LFC and the proposed strategy. Furthermore, the scenarios considered in this study can be summarized as follows:

### Scenario A

Assessing HPG’s performance considering low renewables penetration.

### Scenario B

Assessing HPG’s performance with proposed strategy considering different load variations.

### Scenario C

Assessing HPG’s performance with proposed strategy considering high renewables penetration.

### Scenario D

Assessing the HPG’s performance with proposed strategy considering cyber-attacks and sensitivity analysis.

### Scenario A: assessing the HPG’s performance during low renewables penetration considering SLP

**Fig. 10:**
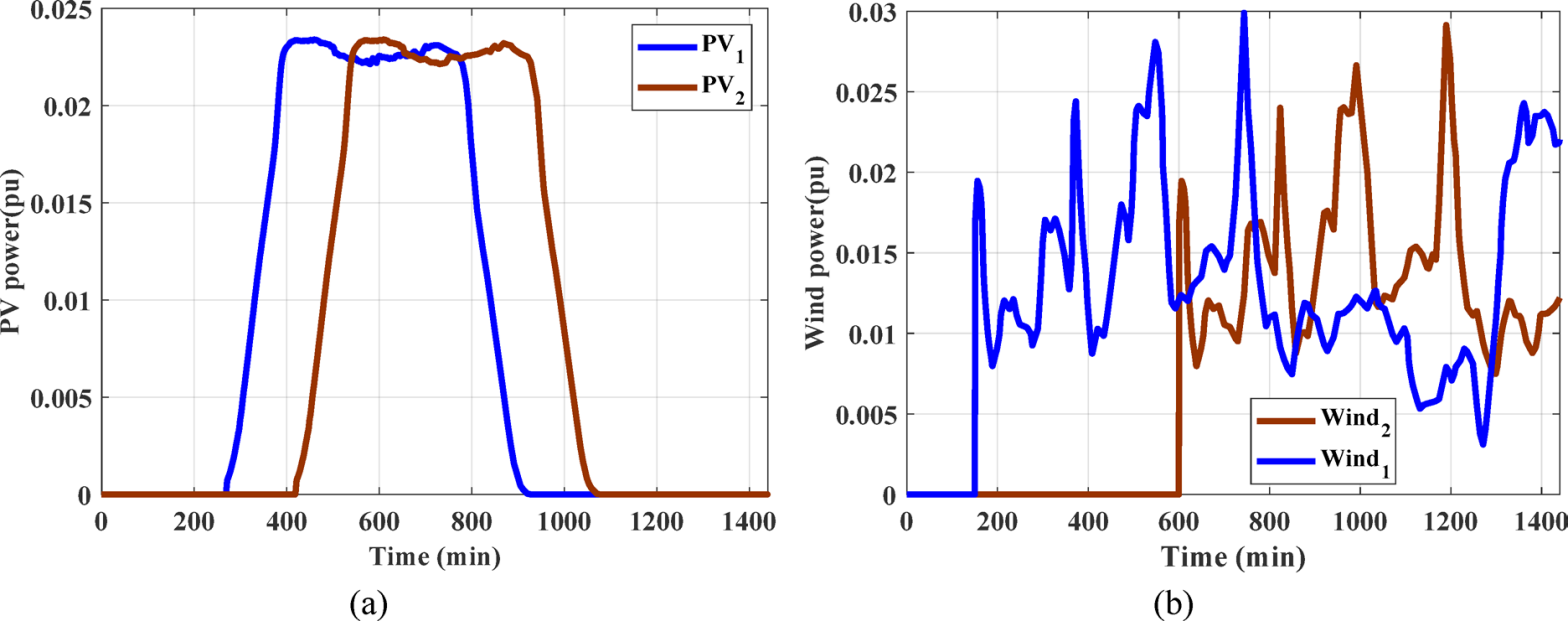
Renewable power considered for low penetration (a) PV power, (b) wind Power.

In this scenario, the response of HPG has been tested considering low RESs penetration. The considered renewable power penetration combines wind and PV power penetration. Figure 10 displays the wind and PV power penetrations considering the start times penetrations of different generation sources. This scenario is divided into two cases; the first one involves confirming the superiority of the EEFO algorithm as well as the PD-PI controller-based LFC. While the second one includes confirming the superiority of the LFC&HESSs-based PD-PI over other strategies.

### Case 1: confirming the superiority of the EEFO algorithm as well as the PD-PI controller in LFC

**Fig. 11:**
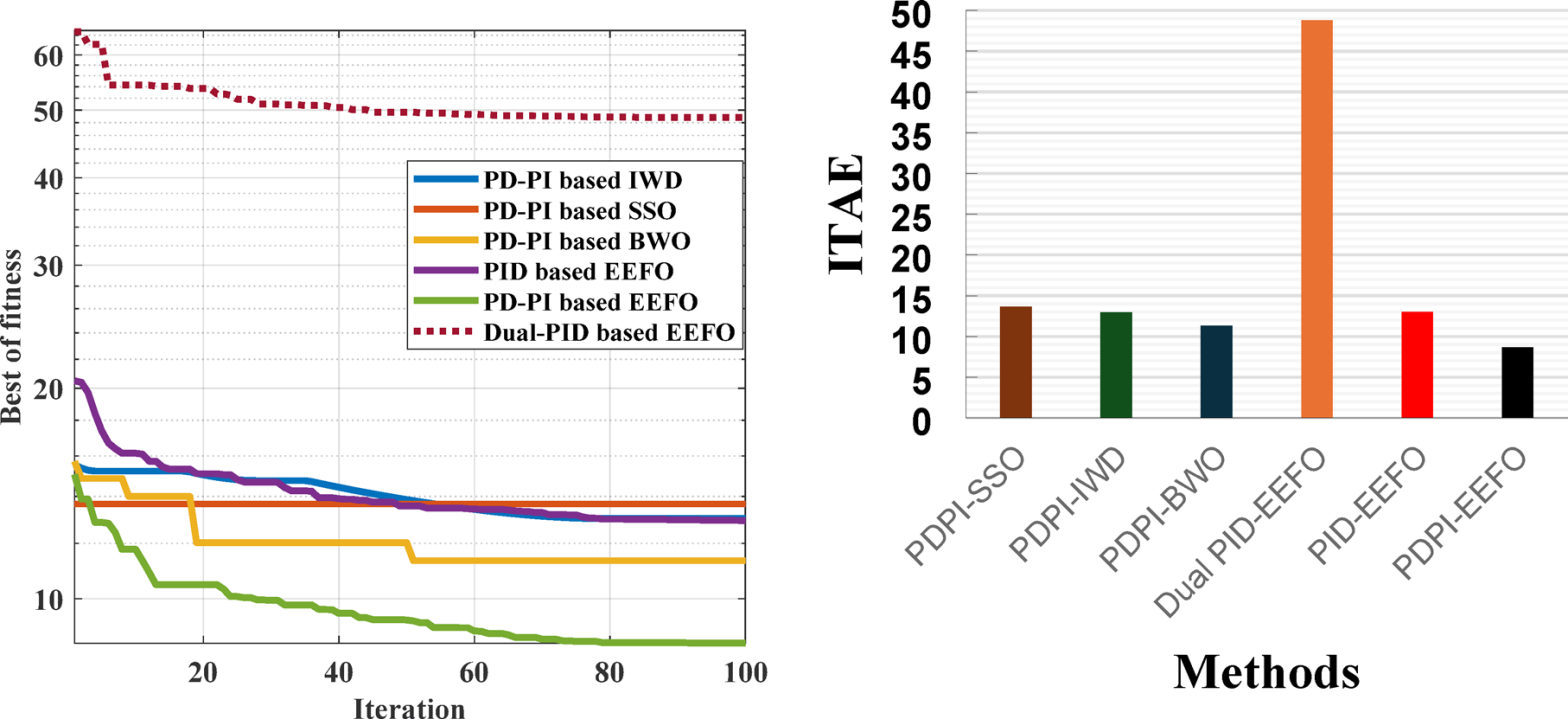
Convergence curves and ITAE bar chart considering low renewable power penetration.

**Fig. 12:**
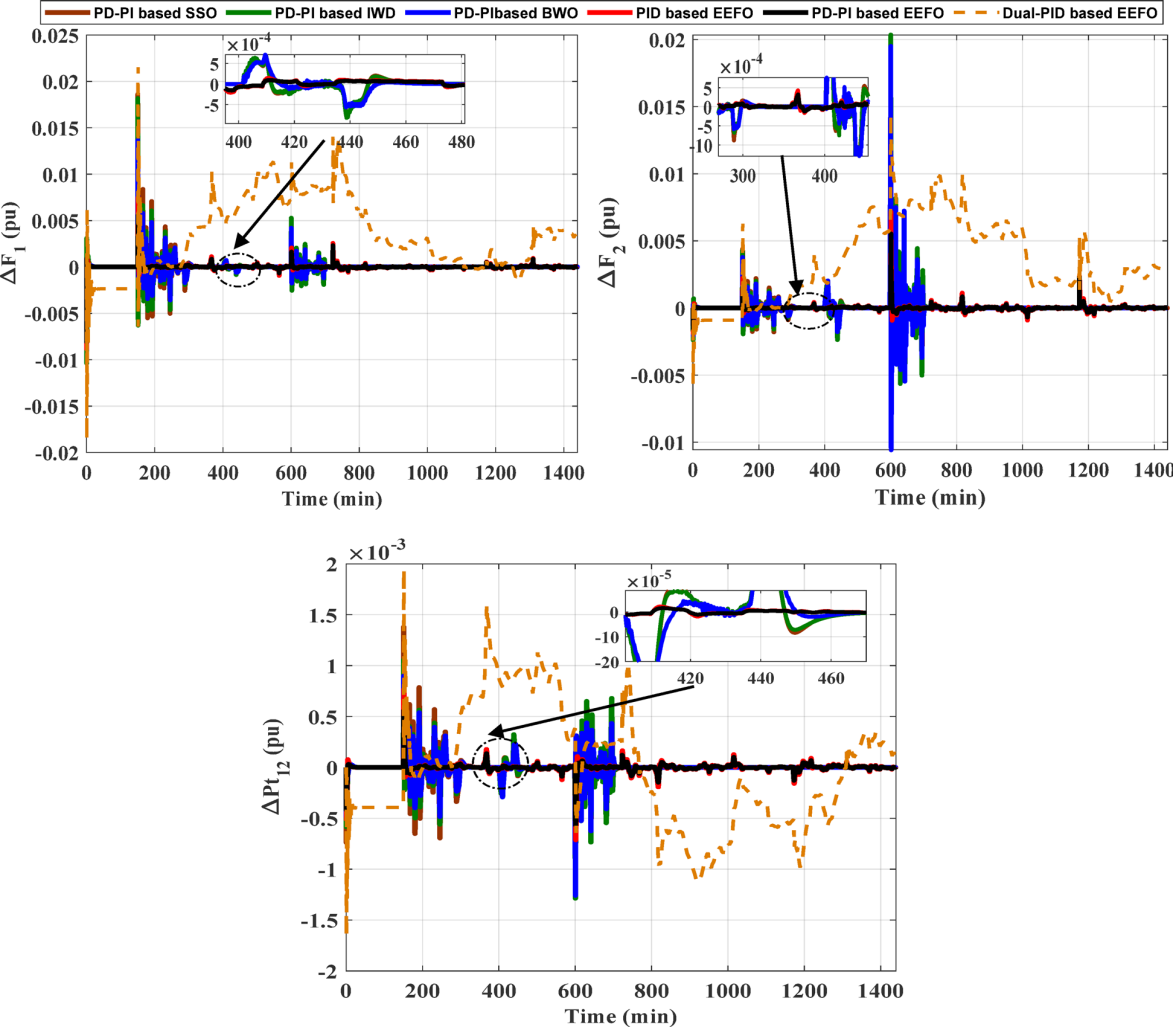
Frequency fluctuation and tie-line power oscillation for case1, scenario A.

The purpose of this case is to validate the superiority of the EEFO algorithm as well as the PD-PI controller in LFC and examine the resilience of the PD-PI controller considering step load deviation (SLD) at area-1 (i.e., Δ P_L_ = 0.01 pu at t = 0 s) of the HPG. The superiority of the EEFO algorithm is validated by comparing its performance with other algorithms such as SSO, IWD and BWO based on the PD-PI controller. The superiority of the PD-PI in LFC is confirmed by comparing its performance with the PID controller. For the simulation process, 100 iterations were performed, with the number of search agents set to 50. The optimal parameters of the considered controllers for this case are tabulated in Table 2. The ITAE and convergence curve in this case is presented in Fig. 11, where the EEFO achieved the lowest error value with the proposed controller compared to other methods. The frequency and the tie-line power responses of the HPG are displayed in Fig. 12. Likewise, Table 3 depicts the numerical values of overshoot (OS) and undershoot (US) for the strategies under study during three different sets of uncertainties. The PD-PI controller relied on the EEFO offers superior improvement in reducing oscillations in frequency deviation as well as exchanged power violation in region-1 and region-2 under step load deviation and low renewable power penetration.

**Table 2:** The optimum gains of the PD-PI and PID controllers in LFC during low renewable penetration.

Alg.	Con.No.	Dual PID			PID			PD-PI			
		K_p_	K_i_	K_d_	K_p_	K_i_	K_d_	K_p1_	K_d_	K_p2_	K_i_
EEFO	C1	9.9946	8.2426	9.5479	9.9739	9.7166	8.1922	9.5870	9.5235	9.9474	9.9718
	C2	0.0017	3.6836	0.7061	9.0106	2.5040	2.9932	9.8263	2.5768	9.9079	9.6726
	C3	5.2286	1.7491	7.3345	9.3976	9.3230	8.1821	9.4867	9.7661	9.3700	9.2886
	C4	9.9812	4.9395	4.9987	9.8834	9.9691	9.9135	9.9117	9.5799	9.7853	9.4924
	C5	9.9542	6.6291	3.3332	8.0110	0.9909	3.8227	9.9752	2.2848	9.8171	4.1358
	C6	9.9913	2.8968	8.1610	9.9989	7.9836	9.7017	9.5980	6.8762	9.6917	9.5869
BWO	C1	-	-	-	-	-	-	9.4915	7.1249	10.0000	7.1882
	C2	-	-	-	-	-	-	9.7062	0.0000	9.5229	3.0203
	C3	-	-	-	-	-	-	10.0000	7.1361	10.0000	0.0000
	C4	-	-	-	-	-	-	10.0000	4.2843	9.5941	4.8689
	C5	-	-	-	-	-	-	10.0000	1.2405	10.0000	0.0000
	C6	-	-	-	-	-	-	10.0000	7.2572	10.0000	7.3231
SSO	C1	-	-	-	-	-	-	7.0605	3.5166	4.3141	5.4701
	C2	-	-	-	-	-	-	5.9490	0.3054	2.9198	2.5181
	C3	-	-	-	-	-	-	5.1805	6.4762	6.9627	5.1442
	C4	-	-	-	-	-	-	7.6903	7.3784	9.1213	6.9253
	C5	-	-	-	-	-	-	4.8448	0.9859	1.2487	7.5195
	C6	-	-	-	-	-	-	6.2735	4.1159	3.1194	2.8150
IWD	C1	-	-	-	-	-	-	8.5827	7.8689	4.3973	4.8506
	C2	-	-	-	-	-	-	9.1858	3.8091	7.8197	4.3120
	C3	-	-	-	-	-	-	10.0000	8.1975	8.0374	8.1958
	C4	-	-	-	-	-	-	3.9358	8.0611	3.7858	4.3294
	C5	-	-	-	-	-	-	7.8342	4.0398	8.6190	8.6430
	C6	-	-	-	-	-	-	8.1969	4.3457	8.1324	7.6283

**Table 3:** Numerical values of OS and US during low renewable penetration for case1, scenario A.

	PD-PI based on SSO algorithm			PD-PI based on IWD algorithm			PD-PI based on BWO algorithm		
	O.S (pu)	U.S (pu)	Ts (s)	O.S (pu)	U.S (pu)	Ts (s)	O.S (pu)	U.S (pu)	Ts (s)
ΔF_1_	0.0186	−0.0104	9.3683	0.0181	−0.0104	11.0564	0.0137	−0.0077	13.0131
ΔF_2_	0.0137	−0.0046	12.6092	0.0203	−0.0056	13.4180	0.0195	−0.0106	16.1036
ΔP_tie_	0.0014	−0.0009	15.1694	0.0011	−0.0013	16.5458	0.0009	−0.0013	19.9467

	Dual PID based on EEFO algorithm			PID based on EEFO algorithm			PD-PI based on EEFO algorithm		
	O.S (pu)	U.S (pu)	Ts (s)	O.S (pu)	U.S (pu)	Ts (s)	O.S (pu)	U.S (pu)	Ts (s)
ΔF_1_	0.0316	−0.0186	NaN	0.00749	−0.00884	6.5584	0.00555	−0.00807	7.3012
ΔF_2_	0.0278	−0.0057	NaN	0.00716	−0.00190	8.3840	0.00552	−0.00154	11.5404
ΔP_tie_	0.0031	−0.0022	NaN	0.00077	−0.00071	12.1233	0.00056	−0.00056	14.4131

**Fig. 13:**
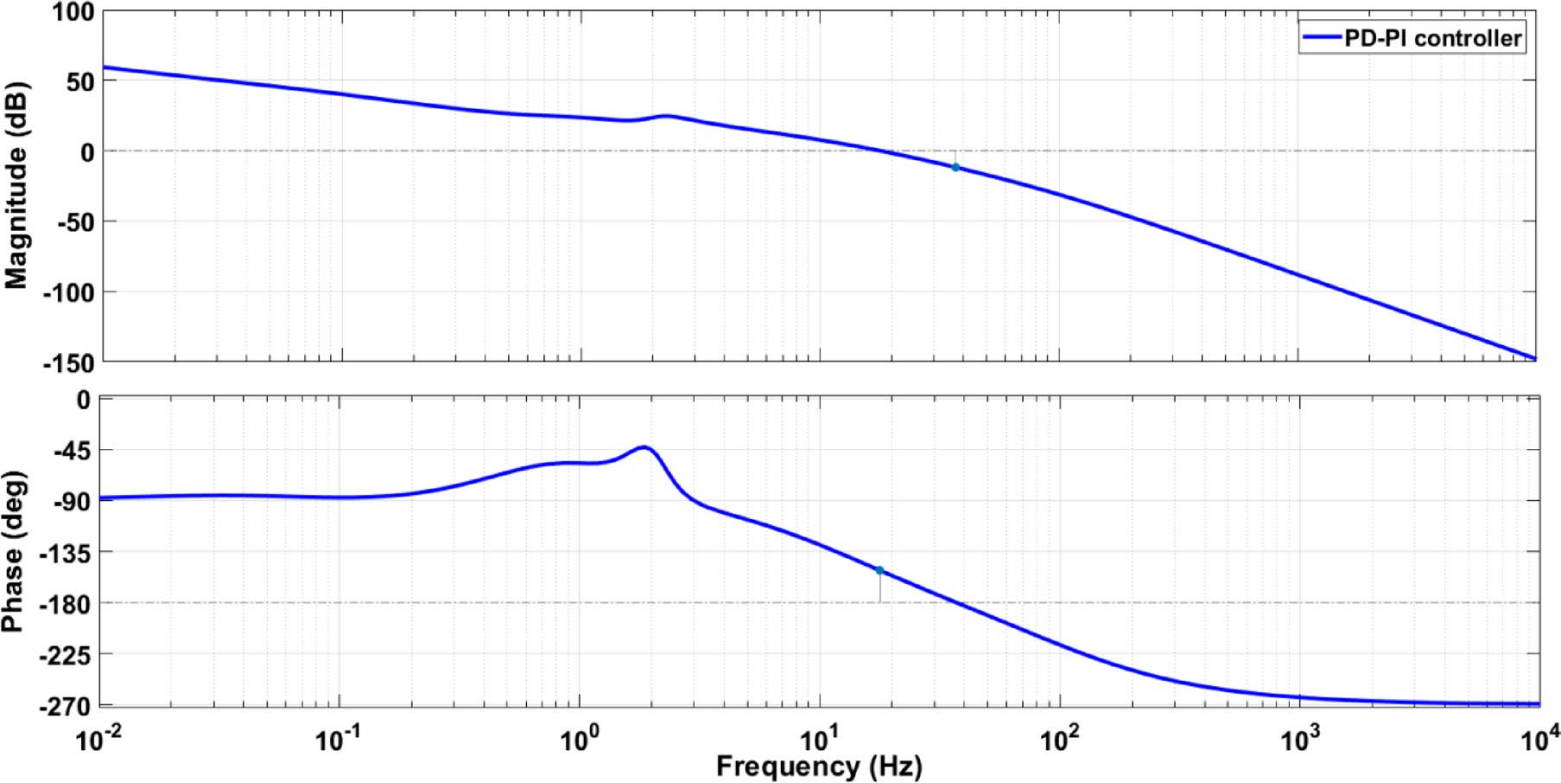
The bode diagram considering the PD-PI based LFC.

Also, the stability of the system with the designed controller via the suggested approach has been assessed. The proposed PD-PI controller’s stability is demonstrated using the bode diagram. Figure 13 displays the bode diagram considering the PD-PI in LFC. The Bode figure demonstrates how the PD-PI controller stability reduces the response at higher frequencies while maintaining strong disturbance rejection capabilities at lower frequencies (greater magnitudes). All things considered, the Bode diagram sheds light on the PD-PI controller’s stability and performance over a variety of frequencies, demonstrating how well it regulates the system’s response through regulated gain and phase shift.

### Case 2: confirming the superiority of the (LFC& HESSs) based PD-PI over other strategies

**Fig. 14:**
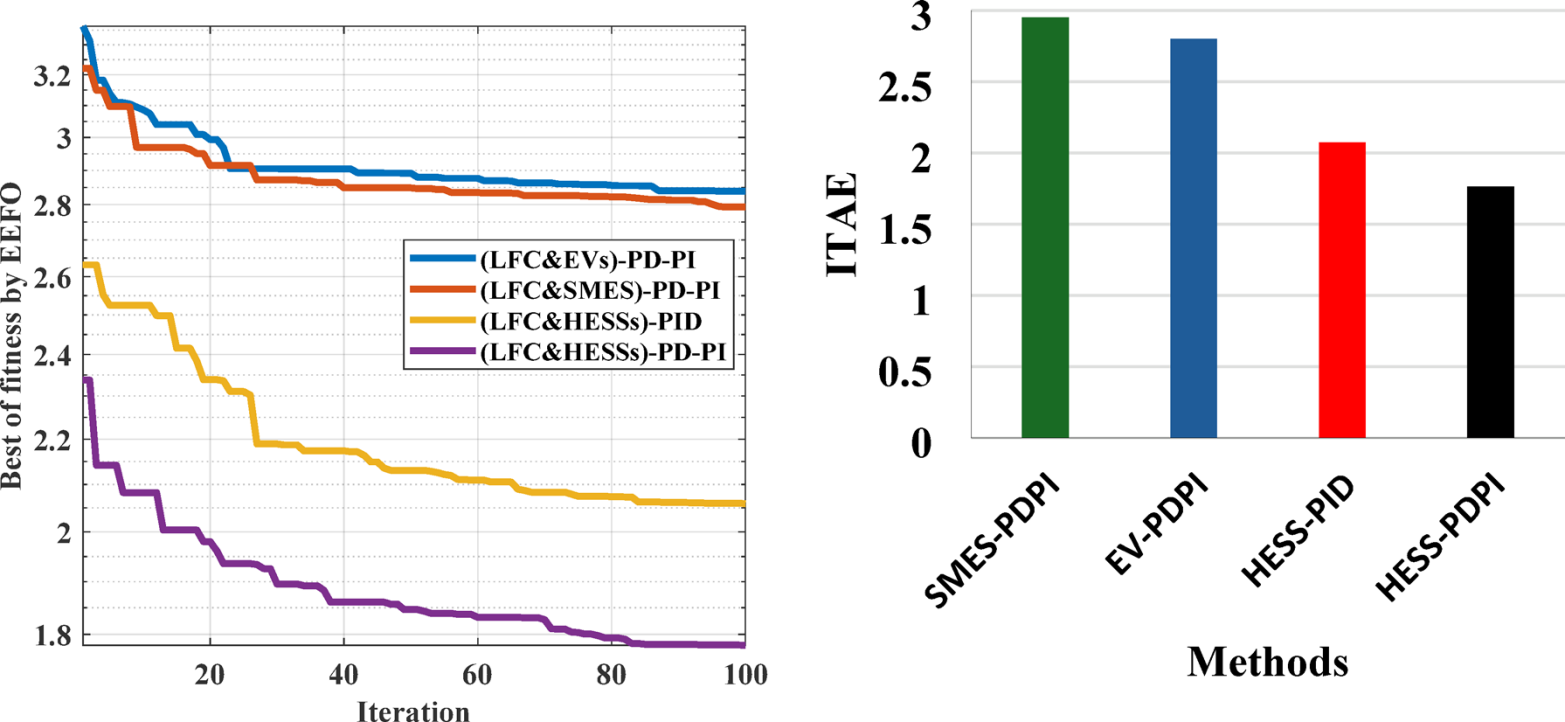
Convergence curve for various methods and ITAE bar chart with ESSs.

**Fig. 15:**
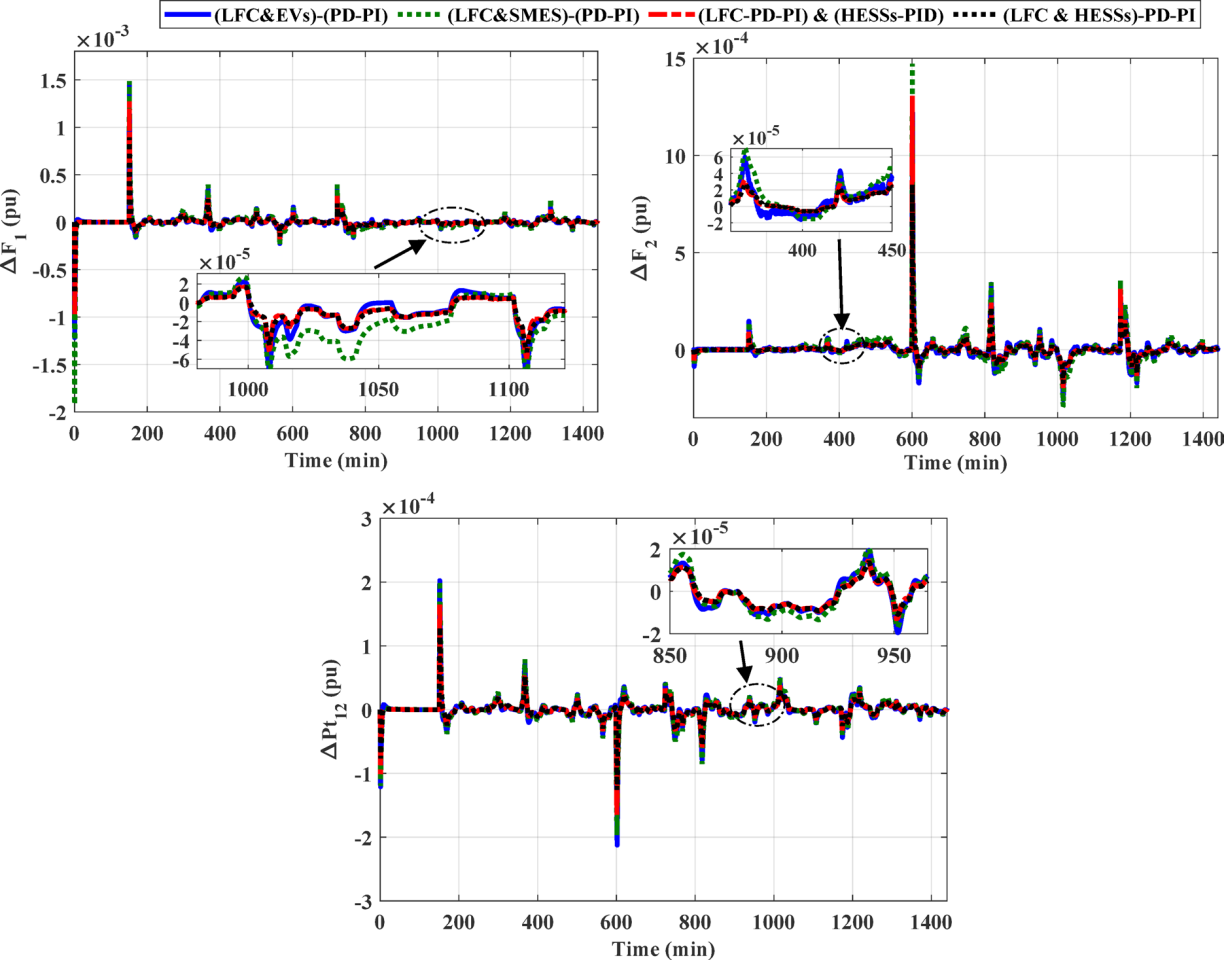
Frequency fluctuation and tie-line power oscillation for case 2, scenario A.

**Fig. 16:**
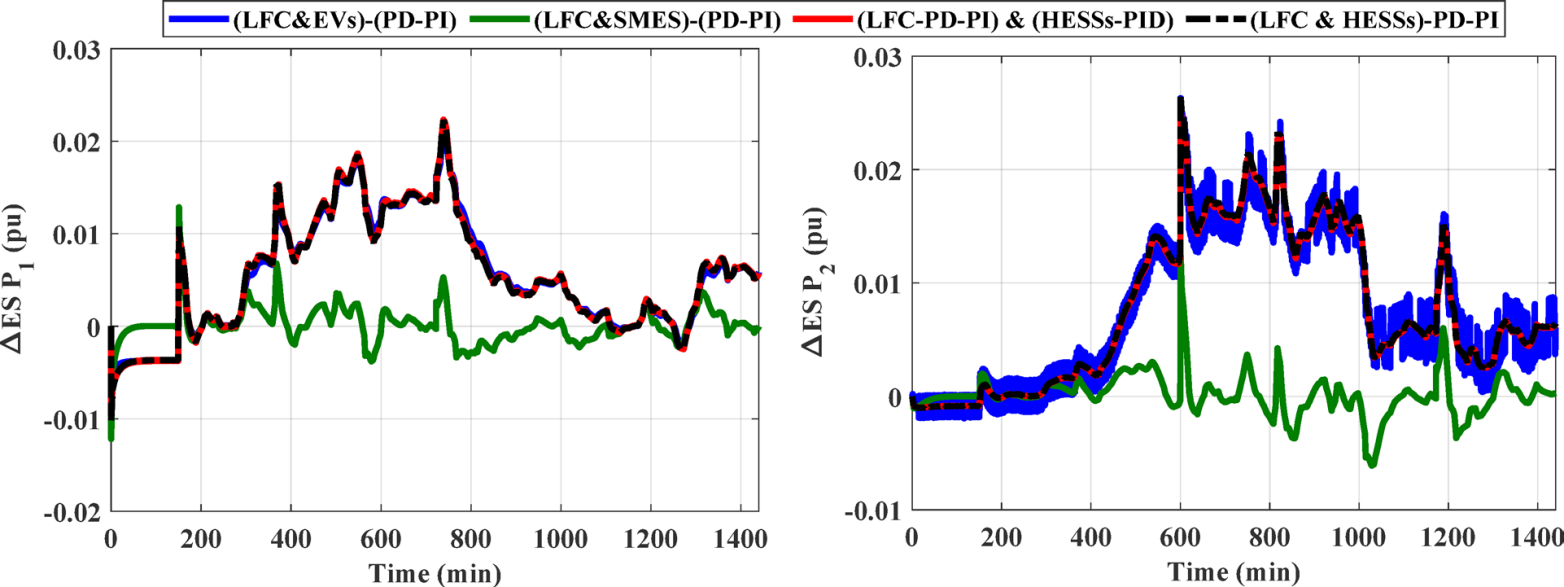
The output power from different strategies for case 2, scenario A.

This case clarifies the superiority of the proposed LFC&HESSs-based PD-PI controller over other strategies like LFC& EVs relied on PD-PI controller, LFC& SMES relied on PD-PI controller, and LFC-PD-PI controller & HESSs -PID controller. The operating conditions is like that used in case 1, scenario A. Furthermore, the optimal parameters of the proposed strategies and others are tabulated in Table 4. The ITAE and convergence curve for various methods with ESSs in this instance is shown in Fig. 14, where the suggested controller produced the EEFO with the lowest error value when compared to different approaches. Additionally, Fig. 15 displays the responses of frequency and the tie-line power violations for case 2, scenario A. For more details, the output power from the strategies considered is shown in Fig. 16. Likewise, Table 5 depicts the numerical values of OS and US for the strategies under study during three different sets of uncertainties. The proposed LFC & HESSs PD-PI controller relied on EEFO offers superior improvement in reducing oscillations in frequency deviation as well as exchanged power violation in region-1 and region-2 under step load deviation and low renewable power penetration.

**Table 4:** The optimal parameters of the approaches considered based on different controllers.

Strategy	ESSs -area	PID			PD-PI			
		K_p_	K_i_	K_d_	K_p1_	K_d_	K_p2_	K_i_
(LFC-&SMESs)	SMES- area-1	-	-	-	9.9206	0.0131	9.9663	9.9951
	SMES- area-2	-	-	-	9.9802	0.4169	9.9953	9.9816
(LFC-&EVs)	EV- area-1	-	-	-	9.7924	0.1860	9.8552	9.9954
	EV- area-2	-	-	-	9.9402	6.0661	9.8796	9.9863
(LFC &HESSs)	SMES- area-1	9.9062	9.9864	0.6378	9.9634	0.3786	6.5281	9.7671
	EV- area-1	9.9976	9.9810	1.2271	9.8885	0.0189	9.9626	9.8212
	SMES- area-1	9.7445	9.7546	0.6289	8.7609	1.4956	9.7284	9.7683
	EV- area-2	9.7738	9.6429	0.0029	9.8347	0.8477	9.7815	9.8103

**Table 5:** Numerical values of OS, Ts (s) and US under three different sets of uncertainties during step load violation.

	LFC & SMES based PD-PI			LFC& EVs based PD-PI		
	O.S (pu)	U.S (pu)	Ts (s)	O.S (pu)	U.S (pu)	Ts (s)
ΔF_1_	0.00148	−0.00192	5.5424	0.00145	−0.00122	11.1101
ΔF_2_	0.00147	−0.00029	45.3003	0.00129	−0.00026	99.9952
ΔP_tie_	0.00020	−0.00020	17.0940	0.00020	−0.00021	19.7868

	LFC &HESSs					
	PID			PD-PI		
	O.S (pu)	U.S (pu)	Ts (s)	O.S (pu)	O.S (pu)	Ts (s)
ΔF_1_	0.00127	−0.00097	3.8754	0.00089	−0.00075	6.1401
ΔF_2_	0.00131	−0.00020	32.5907	0.00085	−0.00017	80.5920
ΔP_tie_	0.00017	−0.00016	5.8302	0.00013	−0.00013	7.4153

### Scenario B: assessing the HPG’s performance with proposed strategy considering different load variations

This scenario is used to examine the resilience of the strategy considered in comparison with other previously mentioned strategies considering different load variations in the presence of low renewable penetration. The operating condition of this scenario is like the operating condition of the previous scenario but considering different load patterns instead of SLP. These patterns of load are intended to imitate real-world variations, allowing for a more comprehensive evaluation of how effectively the approach adapts to changing demands. By examining its performance under these settings, we may find possible strengths and shortcomings in comparison to the other strategies outlined previously. As a result, this scenario is divided into two cases. The first case shows the HPG performance considering series step load variations (SSLV), while the second case considering Random load variations (RLV).

### Case 1: HPG performance considering SSLV.

**Fig. 17:**
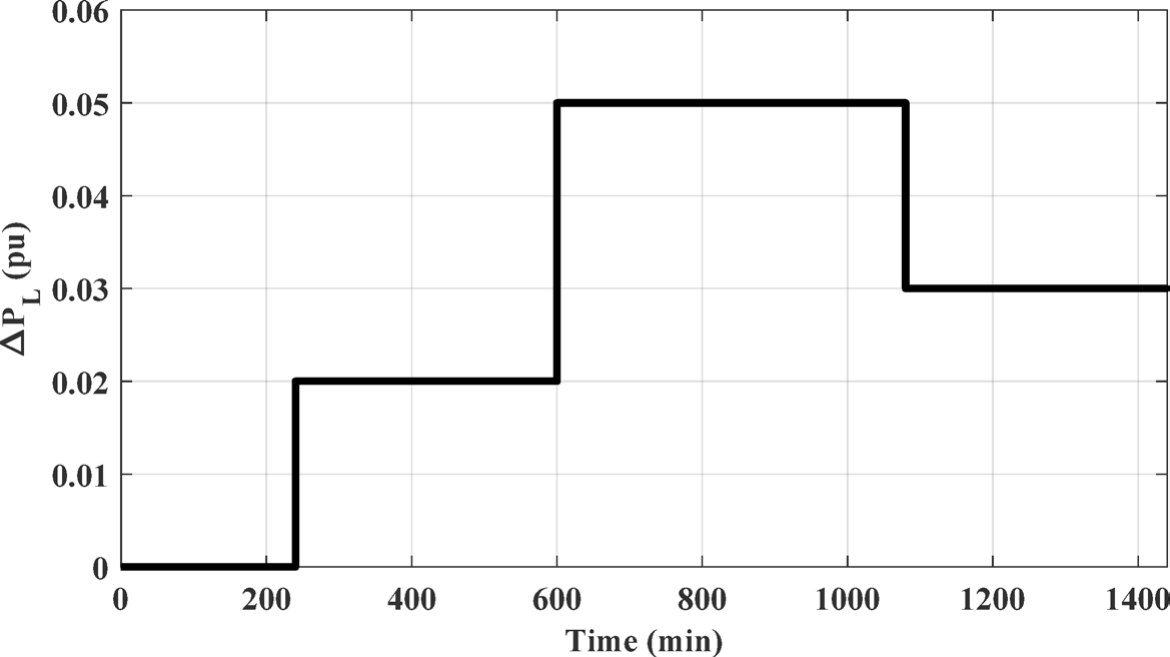
The considered SSLV.

**Fig. 18:**
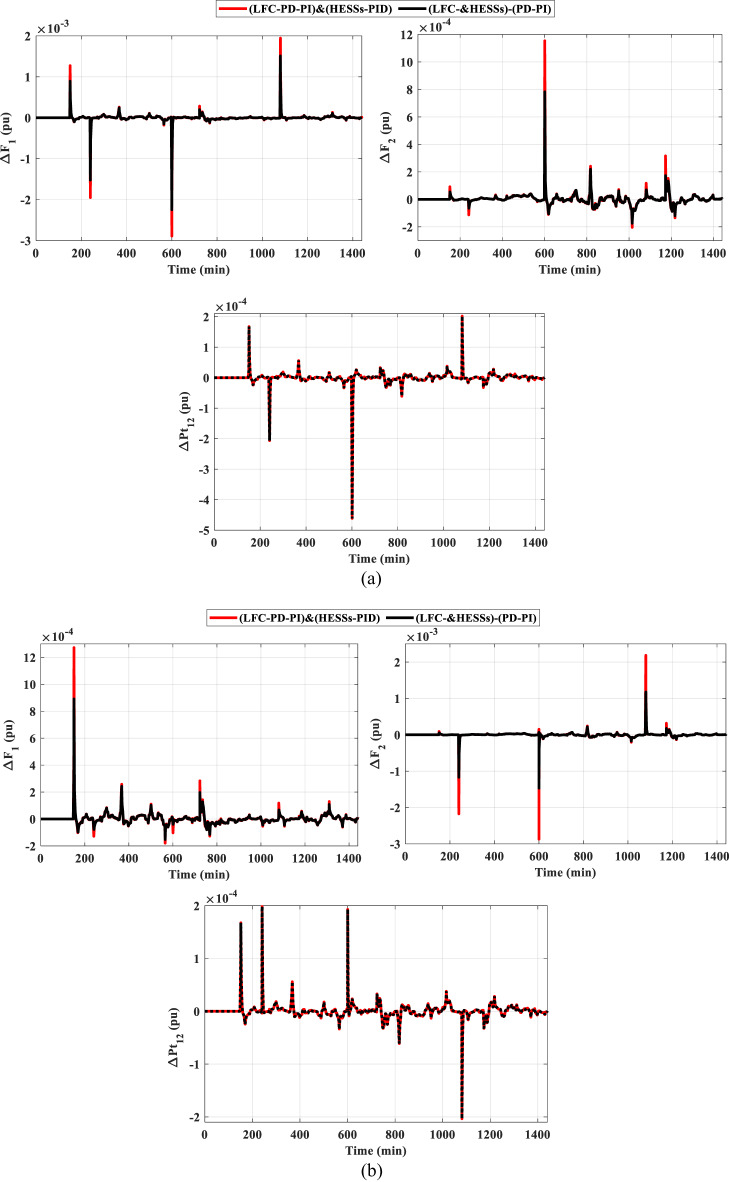
The frequency and the tie-line power responses of HPG case 1, scenario B considering (**a**) SSLV at region-1, and (**b**) SSLV at region-2.

The SSLV is applied to the HPG across all regions. Figure 17 illustrates the SSLV configuration. Initially, the performance of the HPG is evaluated when the SSLV is applied to region (1) Subsequently, the system’s performance is assessed when the SSLV is applied to region (2) The response for this scenario is depicted in Fig. 18. According to Fig. 18, the HESS-based PD-PI controller achieved the lowest overshoot (OS) and undershoot (US) values. For ΔF_1_ and ΔF_2_, the OS values were 0.0015 pu and 0.0008 pu, respectively, while the US values were − 0.00015 pu and − 0.00017 pu, respectively, compared to the HESS-based PID controller. In contrast, the PID controller’s OS values for ΔF_1_ and ΔF_2_ were 0.0019 pu and 0.00115 pu, while its US values were − 0.0029 pu and − 0.0002 pu, respectively. These results demonstrate that, the proposed method delivers superior performance by significantly reducing both OS and US values, confirming its effectiveness in mitigating frequency fluctuations during SSLV. The numerical analysis values of OS, US and Ts during SSLV are tabulated in Table 6.

**Table 6:** Numerical values of OS, US and Ts during SSLV for HPG.

	A1					
	PID			PD-PI		
	O.S (pu)	U.S (pu)	Ts (s)	O.S (pu)	O.S (pu)	Ts (s)
ΔF_1_	0.00194	−0.00289	1370.88	0.00151	−0.00225	1371.10
ΔF_2_	0.00115	−0.00020	1397.84	0.00078	−0.00017	1398.79
ΔP_tie_	0.00020	−0.00046	1371.53	0.00015	−0.00035	1372.11

	A2					
	PID			PD-PI		
	O.S (pu)	U.S (pu)	Ts (s)	O.S (pu)	O.S (pu)	Ts (s)
ΔF_1_	0.00127	−0.00018	1386.73	0.00089	−0.00015	1430.03
ΔF_2_	0.00219	−0.00287	1218.88	0.00118	−0.00147	1397.65
ΔP_tie_	0.00020	−0.00020	1430.94	0.00015	−0.00015	1431.87

### Case 2: HPG performance considering RLV.

**Fig. 19:**
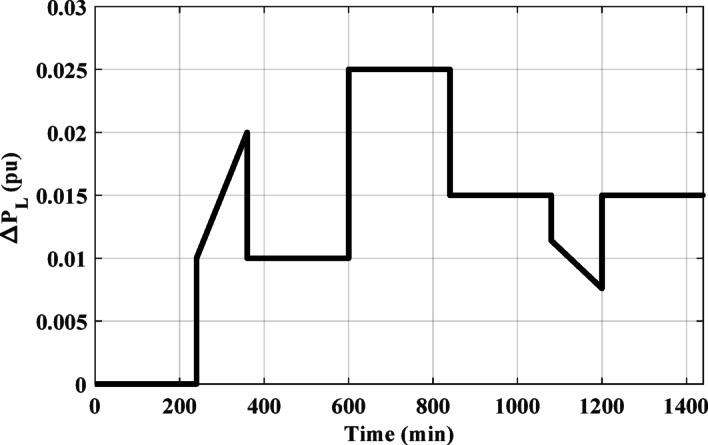
The considered RLV.

**Fig. 20:**
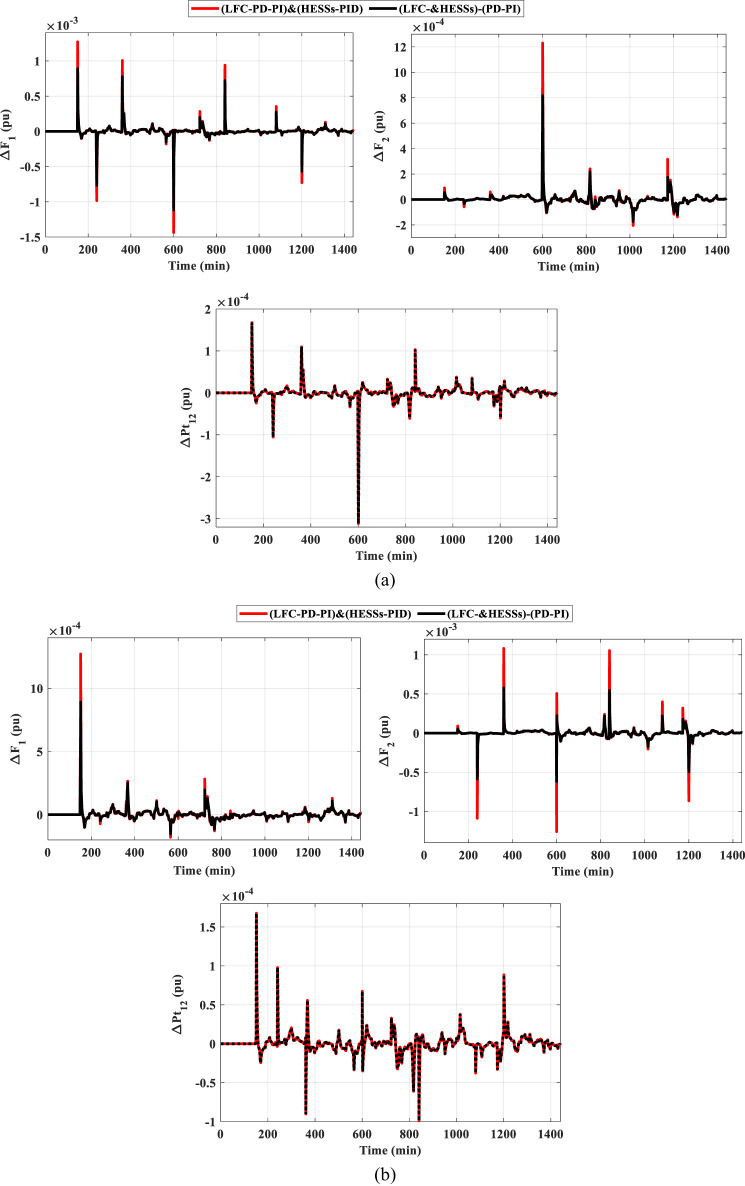
The frequency and the tie-line power responses of HPG case 2, scenario B considering (**a**) RLV at region-1, and (**b**) RLV at region-2.

The RLV is applied to the HPG across all regions. Figure 19 illustrates the RLV configuration. Initially, the performance of the HPG is evaluated when the RLV is applied to region-1. Subsequently, the system’s performance is assessed when the RLV is applied to region-2. The response for this scenario is depicted in Fig. 20. The figure clarifies that, the HESS-based PD-PI technique achieved the lowest values for both OS and US. For ΔF_1_, the OS and US values were 0.0009 pu and − 0.0011 pu, respectively, while for ΔF_2_, they were 0.00081 pu and − 0.00017 pu, respectively, compared to the HESS-based PID controller. Additionally, Fig. 20(b) illustrates the frequency deviations and tie-line power disturbances in region-2, further confirming that the HESS-based PD-PI technique minimized both OS and US values compared to the HESS-based PID. Table 7 displayed the numerical analysis values of OS, US and Ts during RLV. This demonstrates the effectiveness of the proposed strategy in handling load fluctuations and maintaining frequency stability at nominal levels.

**Table 7:** Numerical values of OS, US and Ts during RLV for HPG.

	A1					
	PID			PD-PI		
	O.S (pu)	U.S (pu)	Ts (s)	O.S (pu)	O.S (pu)	Ts (s)
ΔF_1_	0.00127	−0.00144	1386.62	0.00089	−0.00112	1429.72
ΔF_2_	0.00123	−0.00020	1397.78	0.00082	−0.00017	1398.67
ΔP_tie_	0.00017	−0.00031	1429.90	0.00013	−0.00024	1430.84

	A2					
	PID			PD-PI		
	O.S (pu)	U.S (pu)	Ts (s)	O.S (pu)	O.S (pu)	Ts (s)
ΔF_1_	0.00127	−0.00018	1386.76	0.00089	−0.00015	1430.03
ΔF_2_	0.00108	−0.00126	1397.76	0.00058	−0.00062	1399.13
ΔP_tie_	0.00017	−9.90E-05	1431.26	0.00013	−0.00015	1432.24

### Scenario C: assessing the HPG’s performance with proposed strategy considering high renewables penetration

**Fig. 21:**
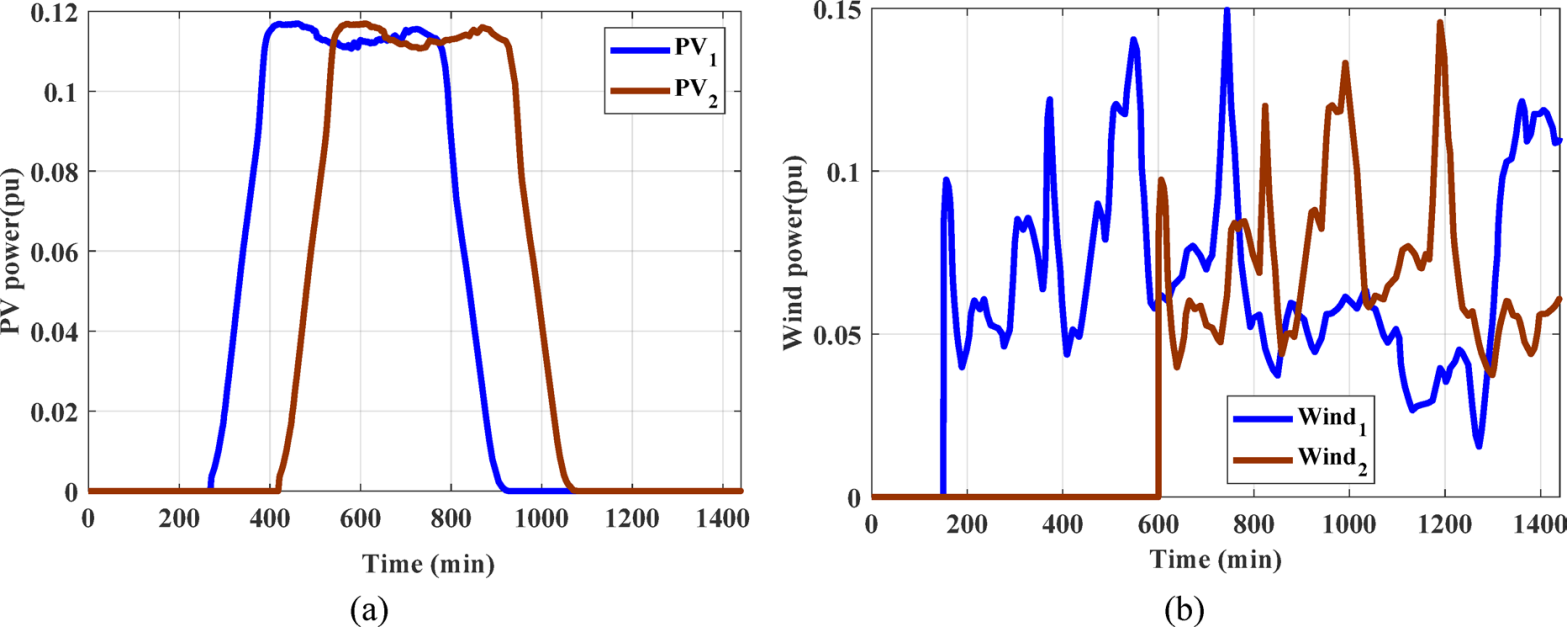
Renewable power considering for high penetration (**a**) PV power (**b**) wind power.

**Fig. 22:**
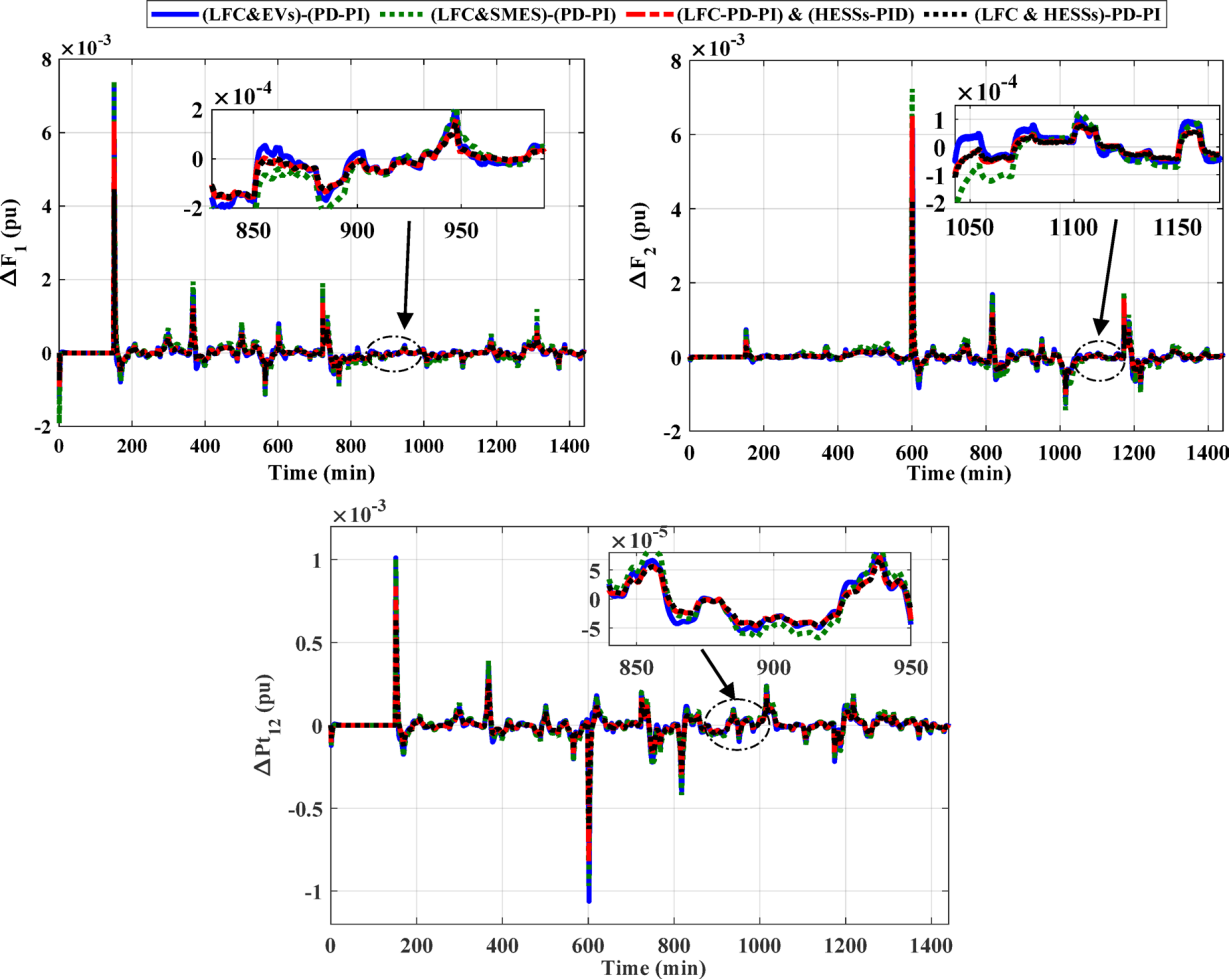
Deviations in frequency and tie-line power change for scenario C considering high renewable penetration.

**Fig. 23:**
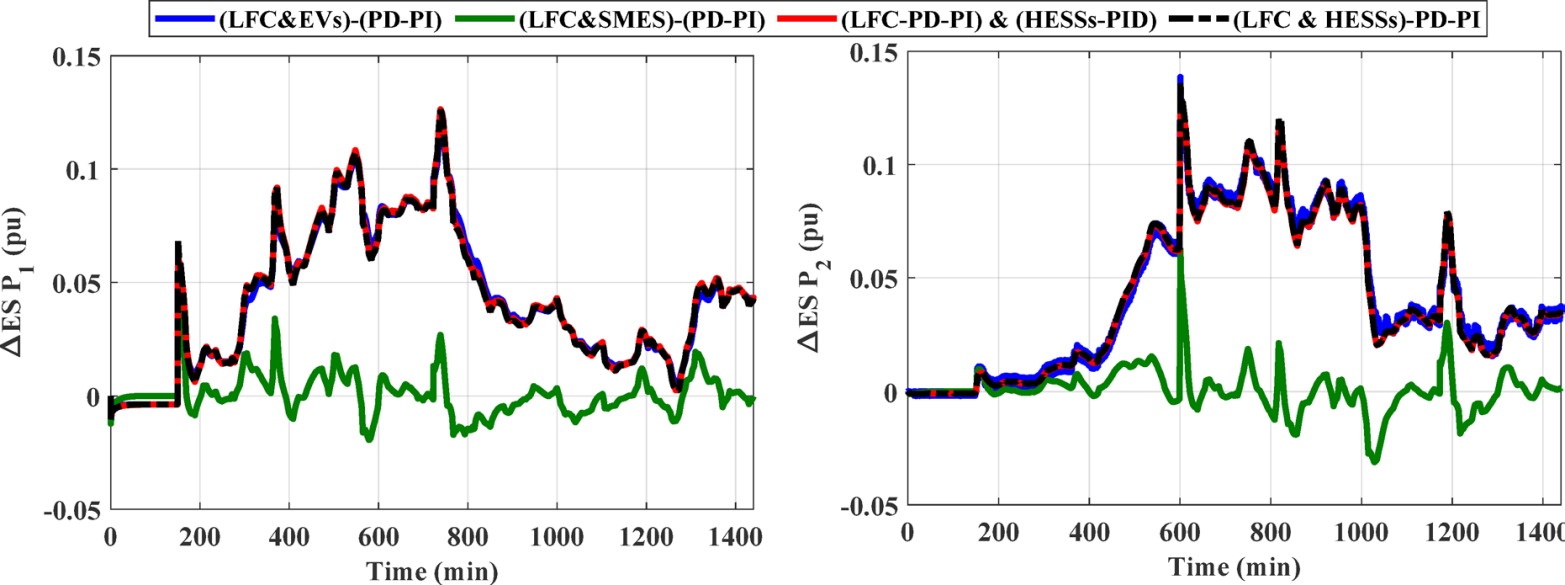
The output power from different strategies for scenario C.

In this scenario, the impact of the high renewables’ energy penetration has been considered. Furthermore, the output power of the RESs for this scenario are displayed in Fig. 21. A SLD on region-1 (i.e., Δ PL = 0.01 pu at t = 0 s) of the considered HPG under study. Figure 22 displays the frequency and the power in tie-line responses for this instance. Similarly, Table 8 depicts the OS and US of the three different strategies for scenario C. Then, the outpower from the strategies considered is shown in Fig. 23. According to the proposed strategy findings based on simulation outcomes as well as numerical measurements, the proposed controller offers better enhancement in mitigating fluctuations of frequency and tie-line power for the constructed grid under SLP and high renewable penetrations.

**Table 8:** Numerical values of OS and US under three different strategies for scenario C.

Response	LFC & SMES based PD-PI		LFC & EVs based PD-PI		LFC& HESSs			
					PID		PD-PI	
	O.S (pu)	U.S (pu)	O.S (pu)	U.S (pu)	O.S (pu)	U.S (pu)	O.S (pu)	O.S (pu)
ΔF_1_	0.00726	−0.00122	0.00743	−0.00192	0.00636	−0.00097	0.00446	−0.00075
ΔF_2_	0.00646	−0.00126	0.00723	−0.00143	0.00650	−0.00102	0.00423	−0.00086
ΔP_tie_	0.00101	−0.00106	0.00101	−0.00097	0.00084	−0.00082	0.00065	−0.00062

### Scenario D: assessing the HPG’s performance considering cyber-attacks and sensitivity analysis

**Fig. 24:**
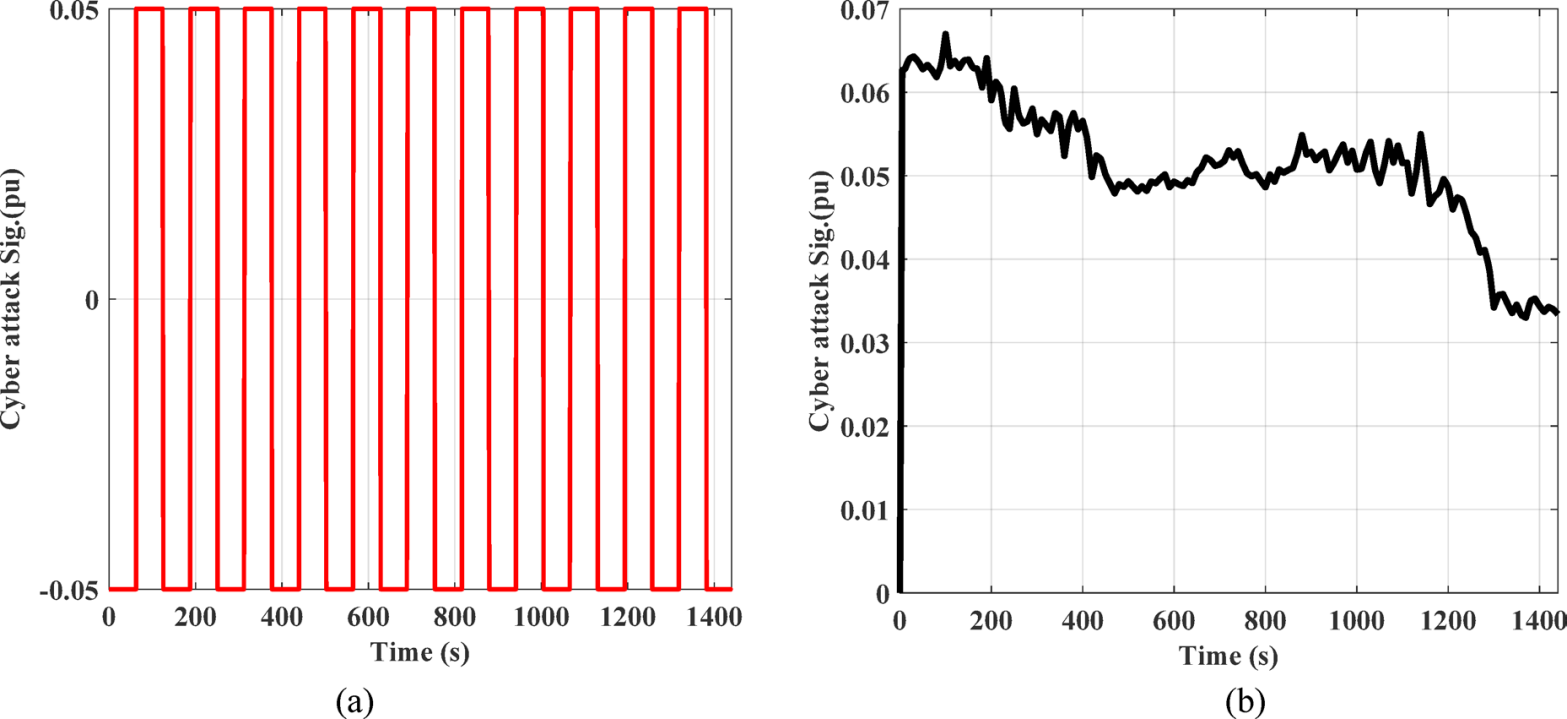
Signals of cyber attacks.

**Fig. 25:**
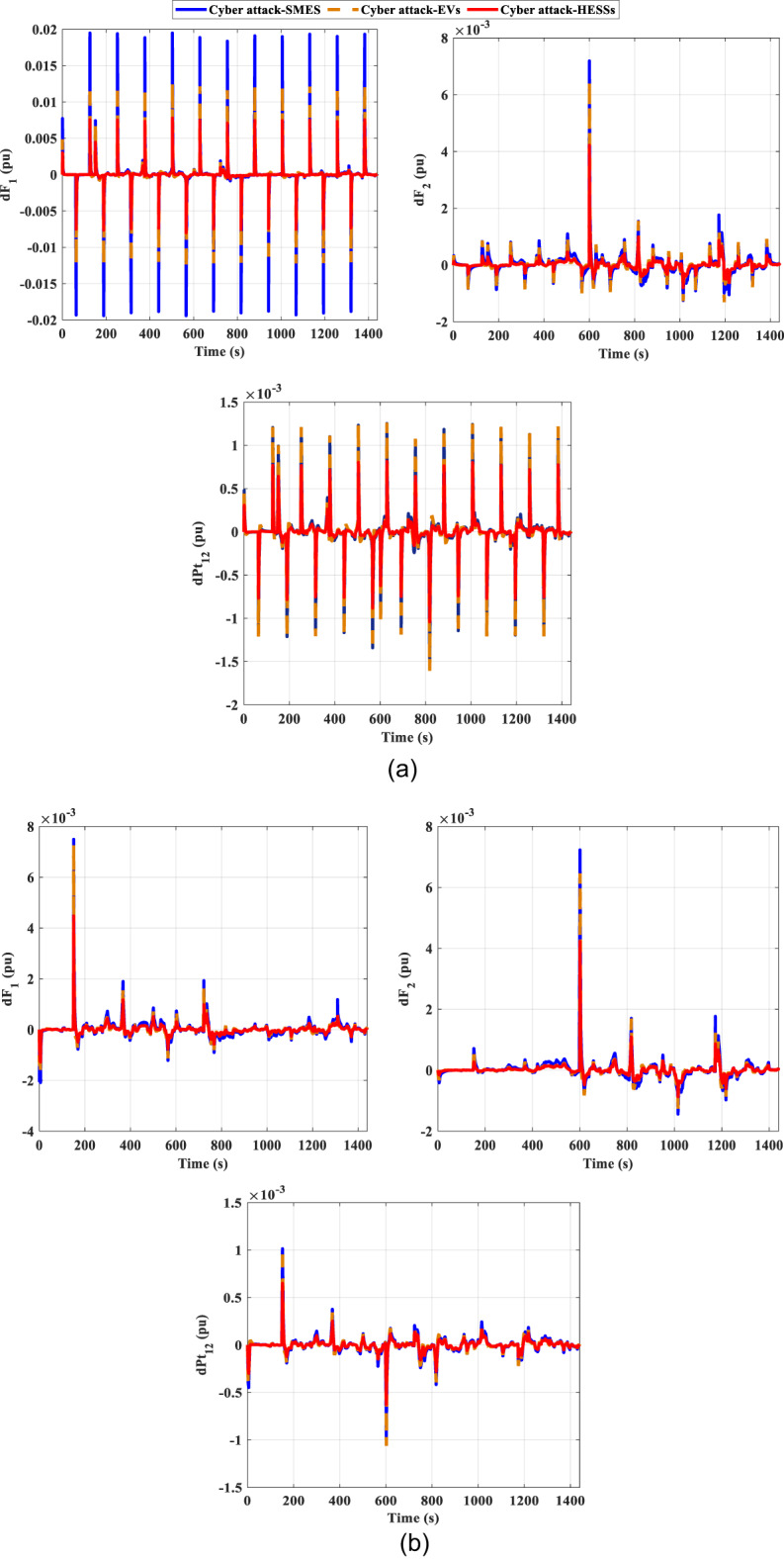
The frequency and the tie-line power responses for scenario D under cyber-attack (**a**) pattern Sig. and (**b**) random Sig.

One of the main problems facing recent smart grids is cyberattacks on LFC. Attackers may interrupt the system’s frequency and transfer of power by altering data or preventing communication. Therefore, detection of intrusions, secure communication, and reliable control mechanisms are crucial for safeguarding LFC systems. In the system under study, two different signals of cyber attacks were introduced to the system as demonstrated in the Fig. 24. Figure 24 (a) represents the attacks from pattren generated, while Fig. 24 (b) denotes the attacks from pattren as random. In addition, the impact of these cyber attacks on the frequency and power deviation in the transmission line is shown in Fig. 25. This shows that random attacks have less of an impact on the system than their counterparts, despite the low frequency deviations, with the highest value of 7.8 E-3 pu and lowest value of −8E-3 pu ​​occurring in the first region.

**Fig. 26:**
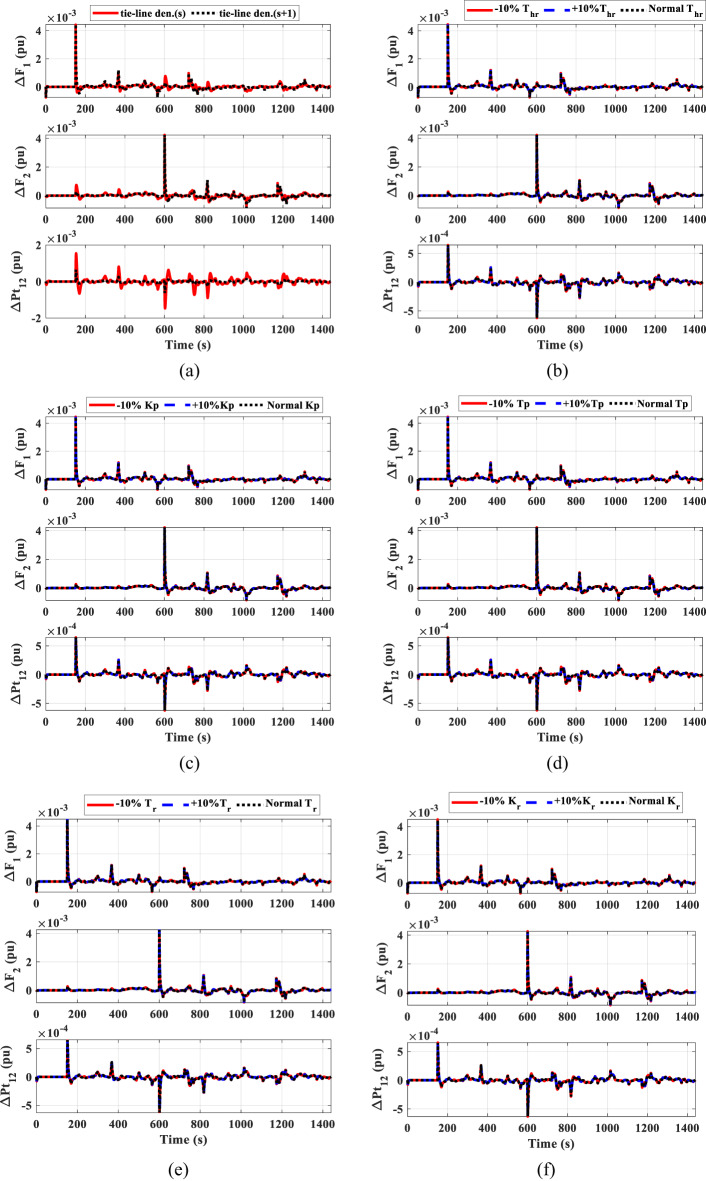
The frequency and the tie-line power responses for scenario C under the uncertainties of the system parameters.

To confirm the superiority and validity of the suggested controller, it is examined by varying the characteristics of the interconnected system. Sensitivity assessment for scenario C is carried out by altering certain system parameters, such as *K*_*p*_, *T*_*p*_, *T*_*hr*_, *T*_*r*_, and *K*_*r*_, at ± 10% of their initial values within HESS. Moreover, Fig. 26 illustrates the effect of under the uncertainties of the system parameters the frequency and power deviation of the transmission lines, which shows no significant change in the deviations in this case compared to the normal case, which confirms the efficiency of the proposed technique. Figure 26 (a) displays the effect of modeling the tie-line power with a denominator (s + 1) instead of (s), which has been shown to reduce the energy deviations in the tie-line.

The most important challenge facing the research is the large simulation time which causes an increase in the time consumed in operation. Finally, the fetched results proved the robustness and competence of the proposed strategy (LFC&HESSs) based on PD-PI controller in vanishing the violations of frequency and tie-line power in a HPG with real renewable energy plants during distinct operating conditions. It can be recommended as a reliable LFC for the interconnected system considered.

## Conclusions

An advanced strategy proposed in this paper effectively addresses the challenges of frequency stability in hybrid interconnected power grids with high renewable energy penetration of real-world RESs. By integrating a hybrid energy storage system (HESSs) combining the long-term balancing capabilities of plug-in electric vehicles (PEVs) and and the rapid response superconducting magnetic energy storage (SMES) units. So, this study bridges the gap between theoretical models and practical applications, offering a realistic representation of modern power grid behavior.The HESSs is controlled using a PD-PI controller whose parameters are optimally tuned via the electric eel foraging optimizer (EEFO). This novel arrangement ensures precise, dynamic, and responsive control to mitigate the inherent fluctuations caused by the variability and intermittency of RESs. Through comprehensive simulation and comparative analyses, the proposed (LFC&HESSs)-based PD-PI controller significantly outperformed traditional methods. Specifically, the PD-PI controller achieved marked improvements over the widely used proportional-integral-derivative (PID) controller, demonstrating faster and more accurate frequency stabilization. When benchmarked against alternative strategies, such as the (LFC& SMES)-based PD-PI controller, (LFC &EVs)-based PD-PI controller, and LFC&HESS-based PID controller, the proposed method achieved performance improvements of 55%, 45%, and 40%, respectively. These results affirm the effectiveness of the proposed strategy as a promising and scalable solution for modern power systems, paving the way for more resilient, adaptable, and sustainable energy grids. In the future, the impact of the suggested strategy will be extended to larger interconnected power systems, particularly focusing on deregulated environments and nonlinear systems. Future studies will also explore integration of alternative energy storage technologies, such as hydrogen electrolyzer systems and redox flow batteries, will be explored especially in systems with high DC voltage. Further investigations will also examine the influence of fault circuits on system stability, providing a more comprehensive understanding of the control strategy’s robustness and effectiveness in diverse, real-world power grid scenarios.

## Supplementary Information

Below is the link to the electronic supplementary material.

Supplementary Material 1

## Data Availability

All data generated or analysed during this study are included in this published article.

## Abbreviations

HESSs: Hybrid Energy Storage Systems
PD-PI: Proportional Derivative-Proportional Integral
PID: Proportional Integral Derivative
PEVs: Plug-in Electric Vehicles
SMES: Superconducting Magnetic Energy Storage
EEFO: Electric Eel Foraging Optimizer
OS: Overshoot
US: Undershoot
RESs: Renewable Energy Sources
LFC: Load Frequency Control
EV: Electrical vehicle
ITAE: Integral Time Absolute Error
ITSE: Integral Time Square Error
BWO: Beluga Whale Optimization
SSO: Sperm Swarm Optimization
IWD: Intelligent Water Drops
TID: Tilt Integral Derivative
PSO: Particle Swarm Optimization
FO: Fractional Order
ACE: Area Control Error
*R*: Turbine regulation constant
*a*_*12*_: The capacity proportion of the control region
*K*_*ps*_: The power system’s gain constant
*B*: The frequency bias coefficient with relation to region
*Tab*: Constant of synchronization
*T*_*ps*_: Time constant for the power system
*K*_*g*_: Gain of the Governor
*K*_*r*_: Gain of the re-heater steam turbine
*K*_*t*_: Gain of the turbine
*K*_*T*_: The degree of involvement in relation to a thermal plant
*T*_*g*_: Constant time for governor
*T*_*r*_: Constant time for re-heater
*T*_*t*_: Constant time for the steam turbine
*K*_*gh*_: Gain of the hydro turbine governor
*T*_*rs*_: The hydro turbine’s speed governor reset time
*T*_*w*_: the water in penstock’s nominal string time
*T*_*gh*_: The hydro turbine’s velocity regulator time constant
*T*_*rh*_: Hydro governor’s reset time constants
*KH*: The involvement portion in relation to a hydroelectric plant
*b*_*g*_: The valve position gas turbine constant
*Y*_*c*_: The time delay constant for the gas turbine’s velocity governor
*T*_*cr*_: The delay in the combustion response of gas turbines
*T*_*cd*_: The volume-time constant of discharge for gas turbine compressors
*c*_*g*_: The gas turbine’s valve positioner
*X*_*c*_: The gas turbine’s delay constant for the velocity governor
*T*_*fc*_: Gas turbine fuel cycle constant
*KG*: The involvement portion in relation to a gas plant
*C*_*nom*_: the nominal values of battery capacity
*R*_*t*_: Transient resistance of EV
*C*_*t*_: Transient capacitance of EV
Min. *SOC*: Minimum battery’s state of charge
*V*_*nom*_: the nominal values of battery voltage
*R*_*s*_: Series resistance of EV
*RT/F*: Constant values
*C*_*batt*_: The power capacity of batteries
Max. *SOC*: Maximum battery’s state of charge
*K*_*smes*_: the SMES control loop’s gain
*K*_*id*_: the time constant of the converter
*Io*: current before a disruption in the load
*T*_*smes*_: The current deviation’s gain adverse feedback
*L*: The SMES coil’s self-inductance
