# Supplementary material for: Advanced control strategy based on hybrid energy storage system for frequency stability of interconnected power system with high renewables penetration
Source: Sci Rep. 2025 Nov 4;15:38483. doi: 10.1038/s41598-025-23283-6 (PMC12586579; doi:10.1038/s41598-025-23283-6)
Supplement: Supplementary file 1 — Supplementary Material 1 [file 41598_2025_23283_MOESM1_ESM.docx]

**Supplementary**

Table S1 Values for the parameters of the two identical regions of the electrical grid under study [11],[31]

|  | **Parameter** | **Value** | **Parameter** | **Value** |
| --- | --- | --- | --- | --- |
| **System** | *R* | 2.4 HZ/MW | *B* | 0.4312 MW/Hz |
|  | *a_12_* | -1 | *Tab* | 0.0433 MW |
|  | *K_ps_* | 68.9566 | *T_ps_* | 11.49 sec |
| **Reheat plant** | *K_g_* | 1 | *T_g_* | 0.08 ses |
|  | *K_r_* | 0.3 | *T_r_* | 10 sec |
|  | *K_t_* | 1 | *T_t_* | 0.3 sec |
|  | *K_T_* | 0.543478 |  |  |
| **Hydro plant** | *K_gh_* | 1 | *T_gh_* | 0.2 sec |
|  | *T_rs_* | 5 sec | *T_rh_* | 28.75 sec |
|  | *T_w_* | 1 sec | *KH* | 0.326084 |
| **Gas plant** | *b_g_* | 0.05 | *c_g_* | 1 |
|  | *Y_c_* | 1 sec | *X_c_* | 0.6 sec |
|  | *T_cr_* | 0.01 sec | *T_fc_* | 0.23 sec |
|  | *T_cd_* | 0.2 | *KG* | 0.130438 |
| **EV** | Penetration levels | 5-10% | *V_nom_* | 364.8 V |
|  | *C_nom_* | 66.2 Ah | *R_s_* | 0.074 Ω |
|  | *R_t_* | 0.047 Ω | *RT/F* | 0.02612 |
|  | *C_t_* | 703.6 F | *C_batt_* | 24.15 KWh |
|  | Min. *SOC* | 10 % | Max. *SOC* | 95 % |
| **SMES** | *K_smes_* | 100 | *T_smes_* | 0.03 sce |
|  | *K_id_* | 0.2 kV/kA | *L* | 0.03 H |
|  | *Io* | 4.5 kA |  |  |
